# Porous Silicon Nanoneedles Efficiently Deliver Adenine Base Editor to Correct a Recurrent Pathogenic *COL7A1* Variant in Recessive Dystrophic Epidermolysis Bullosa

**DOI:** 10.1002/adma.202414728

**Published:** 2025-03-12

**Authors:** Salman Ahmad Mustfa, Marija Dimitrievska, Cong Wang, Chenlei Gu, Ningjia Sun, Katarzyna Romańczuk, Pawel Karpinski, Łukasz Łaczmański, John A. McGrath, Joanna Jacków‐Malinowska, Ciro Chiappini

**Affiliations:** ^1^ Centre for Craniofacial and Regenerative Biology King's College London London SE1 1UL UK; ^2^ St John's Institute of Dermatology School of Basic & Medical Biosciences King's College London London SE1 1UL UK; ^3^ London Centre for Nanotechnology King's College London London SE1 1UL UK; ^4^ Hirszfeld Institute of Immunology and Experimental Therapy Polish Academy of Sciences Wroclaw 53‐114 Poland; ^5^ Department of Genetics Wroclaw Medical University Wroclaw 50‐367 Poland

**Keywords:** advanced therapy, cell therapy, gene editing, nanoneedles, porous silicon

## Abstract

Base editing, a CRISPR‐based genome editing technology, enables precise correction of single‐nucleotide variants, promising resolutive treatment for monogenic genetic disorders like recessive dystrophic epidermolysis bullosa (RDEB). However, the application of base editors in cell manufacturing is hindered by inconsistent efficiency and high costs, contributed by suboptimal delivery methods. Nanoneedles have emerged as an effective delivery approach, enabling highly efficient, non‐perturbing gene therapies both in vitro and in vivo. Here we demonstrate that nanoneedle delivery of an adenine base editor corrects a heterozygous single‐nucleotide pathogenic variant in *COL7A1* in primary RDEB fibroblasts in vitro with 96.5% efficiency, without inducing off‐target variants. The nanoneedle delivery maintains cell viability and displays modest phenotypical alterations unlike conventional cationic lipid transfection. The nanoneedle‐mediated editing significantly increases the production and secretion of full‐length type VII collagen protein, contributing to restore functional fibroblasts phenotype by improving cell adhesion. These findings underscore the suitability and safety of nanoneedles for gene editing in a clinically relevant context of cell manufacturing, establishing a foundation for their use in cell therapies.

## Graphical abstract

Base editing using nanoneedles efficiently corrects pathogenic variants in the type VII collagen gene of primary fibroblasts from patients with recessive dystrophic epidermolysis bullosa. Nanoneedle editing induces minimal and reversible transcriptional perturbations while restoring the production and secretion of functional type VII collagen. The study highlights the value of nanoneedles as a tool for base editing in cell therapies for genetic disorders.

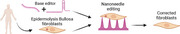

## 1 Introduction

Recessive dystrophic epidermolysis bullosa (RDEB) is a genetic skin fragility disorder caused by loss‐of‐function variants in the *COL7A1* gene encoding type VII collagen (C7), which is the main component forming anchoring fibrils at the dermal‐epidermal junction.^[^ ^1^, ^2^ ^]^ These anchoring fibrils provide structural stability, and pathogenic variants in *COL7A1* disrupt fibril assembly, leading to weakened skin integrity. As a result, individuals with RDEB are prone to severe blistering and wounding of the skin and mucous membranes from minor mechanical stress.^[^ ^3^, ^4^ ^]^

The recent development of beremagene geperpavec (B‐VEC) marks a significant milestone in the treatment of RDEB, being both the first FDA‐approved therapy for the condition and the first topical gene therapy available. Using a herpes simplex virus‐based vector B‐VEC delivers functional *COL7A1* genes, restoring C7 production in vivo^[^ ^5^ ^]^ On the other hand, cell therapies promise long‐term improvement of the RDEB disease phenotype. Clinical studies using *COL7A1* integrative viral vectors demonstrated grafting of autologous, epidermal sheets^[^ ^6^ ^]^ and intradermal injections of autologous fibroblasts.^[^ ^7^ ^]^ CRISPR‐derived gene editing systems are a valuable tool to support autologous cell therapies, demonstrating pre‐clinical evidence of efficient and precise correction of pathogenic variants.^[^ ^8^, ^9^ ^]^ In particular, base editors enable the precise and efficient introduction of single nucleotide variants without causing double‐strand breaks (DSBs). Adenine base editors (ABEs) specifically convert A•T base pairs into G•C. A few versions of ABEs have been developed, with ABE7.10 being the first and ABE8e offering improved efficiency and specificity. Indeed, pre‐clinical studies show that ABEs can efficiently correct selected RDEB pathogenic variants in *COL7A1* restoring C7 expression, presenting a potential therapeutic option.^[^ ^10^ ^]^ Compared to other CRISPR systems that depend on DSBs and homology‐directed repair, base editing is a safer and more refined approach to correct a variant without insertion or deletions at the target site, and lower off‐target effects.

However, the delivery of base editing systems to primary cells such as RDEB dermal fibroblasts remains challenging as existing approaches such as electroporation, viral vectors, and lipid nanoparticles yield a combination of low transfection efficiency, immunogenicity and/or safety concerns.^[^ ^11^ ^]^ Porous silicon (pSi) nanoneedles are arrays of conical nanostructures that have emerged as an efficient delivery system for nucleic acids.^[^ ^12^ ^]^ pSi is a highly biocompatible material, highly manufacturable^[^ ^13^, ^14^, ^15^, ^16^ ^]^ and bioresorbable under physiological conditions due to its high porosity and surface‐to‐volume ratio.^[^ ^17^, ^18^ ^]^ pSi has a favorable toxicology profile,^[^ ^19^, ^20^ ^]^ an exceptionally high drug loading capacity^[^ ^21^, ^22^ ^]^ and the ability to control payload release with desirable kinetics.^[^ ^20^, ^23^ ^]^ Nanoneedles can access the cytoplasm when interfaced with cells to efficiently deliver the cargo with minimal cell perturbations.^[^ ^24^ ^]^ This direct intracellular access underpins nanoneedles’ ability to deliver labile biomolecules, through a process known as nanoinjection.^[^ ^25^ ^]^ Nanoneedles can also be used for biosensing of intracellular molecules given their high surface area to volume ratio, which enhances loading capacity.^[^ ^26^, ^27^, ^28^ ^]^ A first‐of‐its‐kind study utilised nanoinjection for gene therapy showing that rapid nanoneedle application could deliver plasmids to skin and skeletal muscle cells, efficiently inducing neovascularization in vivo, showing that nanoinjection does not elicit cell death, significant phenotypical alterations, or an observable, acute or chronic immune response.^[^ ^12^ ^]^ Strategies exist to integrate nanoneedles within medical devices, including skin bandages, contact lenses and catheters. Such flexible devices can be used to interface with various tissues for ex vivo or in vivo delivery.^[^ ^29^, ^30^ ^]^ In particular, nanoneedles incorporated within skin wound bandages that are routinely used for RDEB wound care have demonstrated efficient topical injection of nucleic acids with minimal discomfort in a mouse model.^[^ ^31^ ^]^ Owing to this capability, nanoinjection can support genetic engineering,^[^ ^32^ ^]^ including nucleic acid therapies in human tissues,^[^ ^33^ ^]^ and in vitro delivery of CRISPR‐Cas9.^[^ ^34^ ^]^ However, to establish nanoneedles’ suitability for genetic engineering in cell manufacturing, nanoinjection must still demonstrate effective gene editing in primary human cells within a disease context, along with evidence of functional phenotype recovery following the correction of hereditary genetic variants.

**Figure 1:**
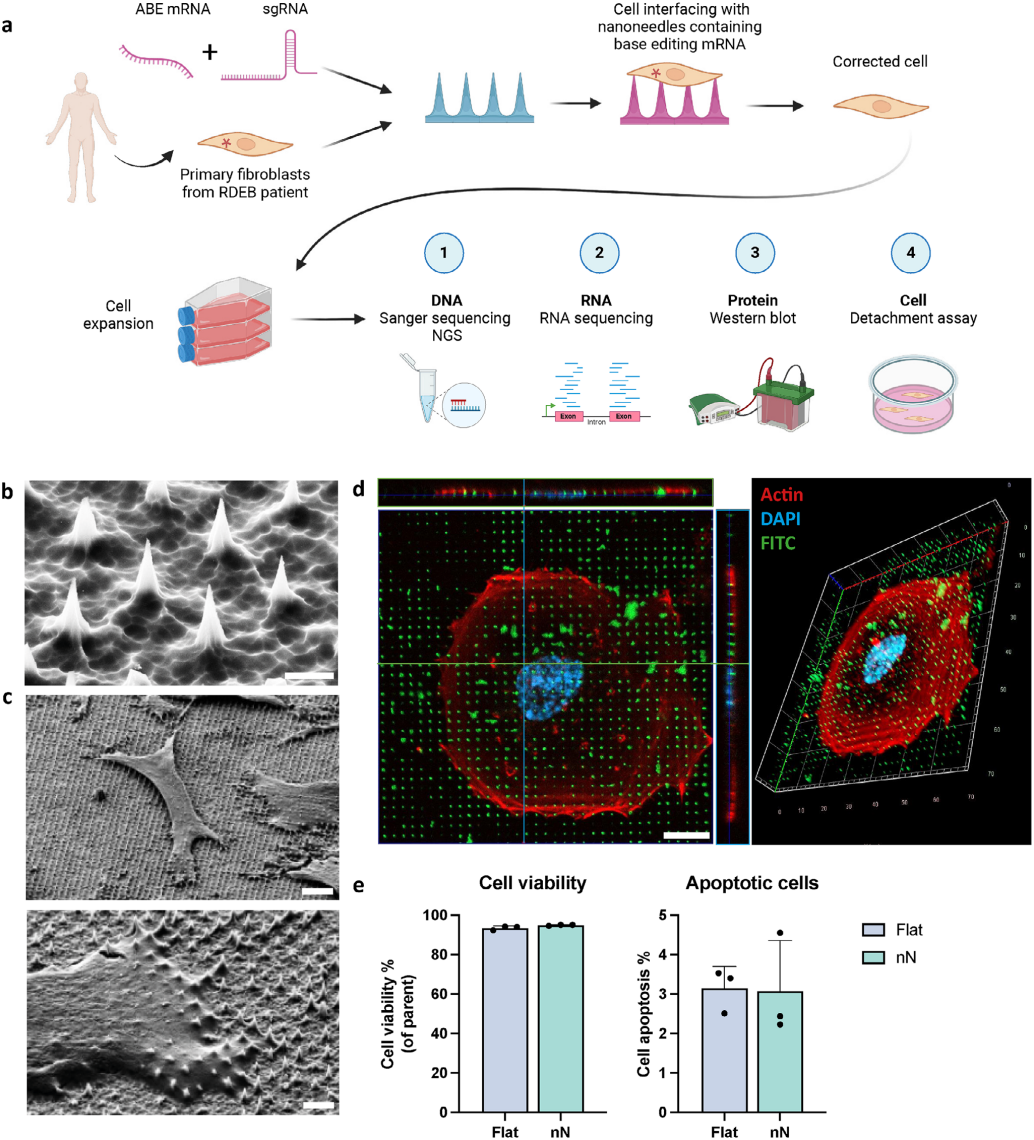
The application of nanoneedles to patient‐derived primary RDEB fibroblasts. a) Schematic of the nanoneedle editing strategy for primary RDEB fibroblasts and the downstream analysis to assess gene editing effects assessing genomic and transcriptomic editing, collagen type VII protein production, and cell adhesion. Created in BioRender. b) Scanning electron micrograph showing nanoneedle morphology. Scale Bar, 1 µm. c) Scanning electron micrographs of primary RDEB fibroblasts over nanoneedles. Scale Bar, 10 µm (top), 5 µm (bottom). d) Orthogonal projections and 3D visualization of a confocal microscopy z‐stack showing primary RDEB fibroblasts growing on FITC labeled nanoneedles. Scale Bar, 10 µm. e) Quantification of viability and caspase‐3/7 activity by flow cytometry for cells grown on a flat silicon or nanoneedle chip for 24 h. Data are presented as mean values ± S.D. Statistical significance was determined using the Mann–Whitney test. N = 3 independent biological samples.

In this study, we developed a nanoinjection approach for base editing (nanoneedle editing) in vitro and applied it to correct a single nucleotide pathogenic variant (c.5047 C>T in *COL7A1*) causing RDEB in patient‐derived primary fibroblasts (**Figure** **1**a). We showed that nanoinjection induced only minor changes in the transcriptomic profile, unlike cationic lipid transfection. We performed nanoneedle editing in fibroblasts, achieving >90% efficiency for the *COL7A1* variant correction rate with no detectable off‐target editing. Nanoneedle editing restored the production of C7 leading to an improvement in RDEB phenotype. These findings demonstrated nanoneedle editing in epidermolysis bullosa as a model disease, laying the groundwork for the use of nanoinjection for CRISPR‐based gene editing in cell therapies.

## 2 Results

### 2.1 Characterisation of Primary RDEB Fibroblast Interfacing with Porous Silicon Nanoneedles

We first assessed nanoneedle cytocompatibility upon interfacing with the primary RDEB fibroblasts carrying the c.5047 C>T heterozygous compound pathogenic variant of interest. We used 1.2–1.5 µm tall nanoneedles with a sharp tip (80 nm) to facilitate efficient transfection. Within 24 h of seeding, the fibroblasts appeared to interface with and grow on the nanoneedles, showing characteristic elongation and spreading (Figure 1b,c). Confocal microscopy confirmed an efficient nanoneedle interfacing across the whole cell population (Figure 1d). To confirm the cytocompatibility of nanoinjection, we quantified the fraction of dead cells by propidium iodide staining and apoptotic cells by caspase‐3/7 activation using flow cytometry (Figure 1e). Cell on nanoneedles displayed comparable viability to those on flat silicon chips (94.9% and 93.5%, respectively) and physiological levels of apoptosis (3.1% and 3.1%, respectively), indicating that nanoneedle interfacing is cytocompatible with primary RDEB fibroblasts.

### 2.2 Nanoinjection of Base Editor has Minimal Impact on Gene Expression

**Figure 2:**
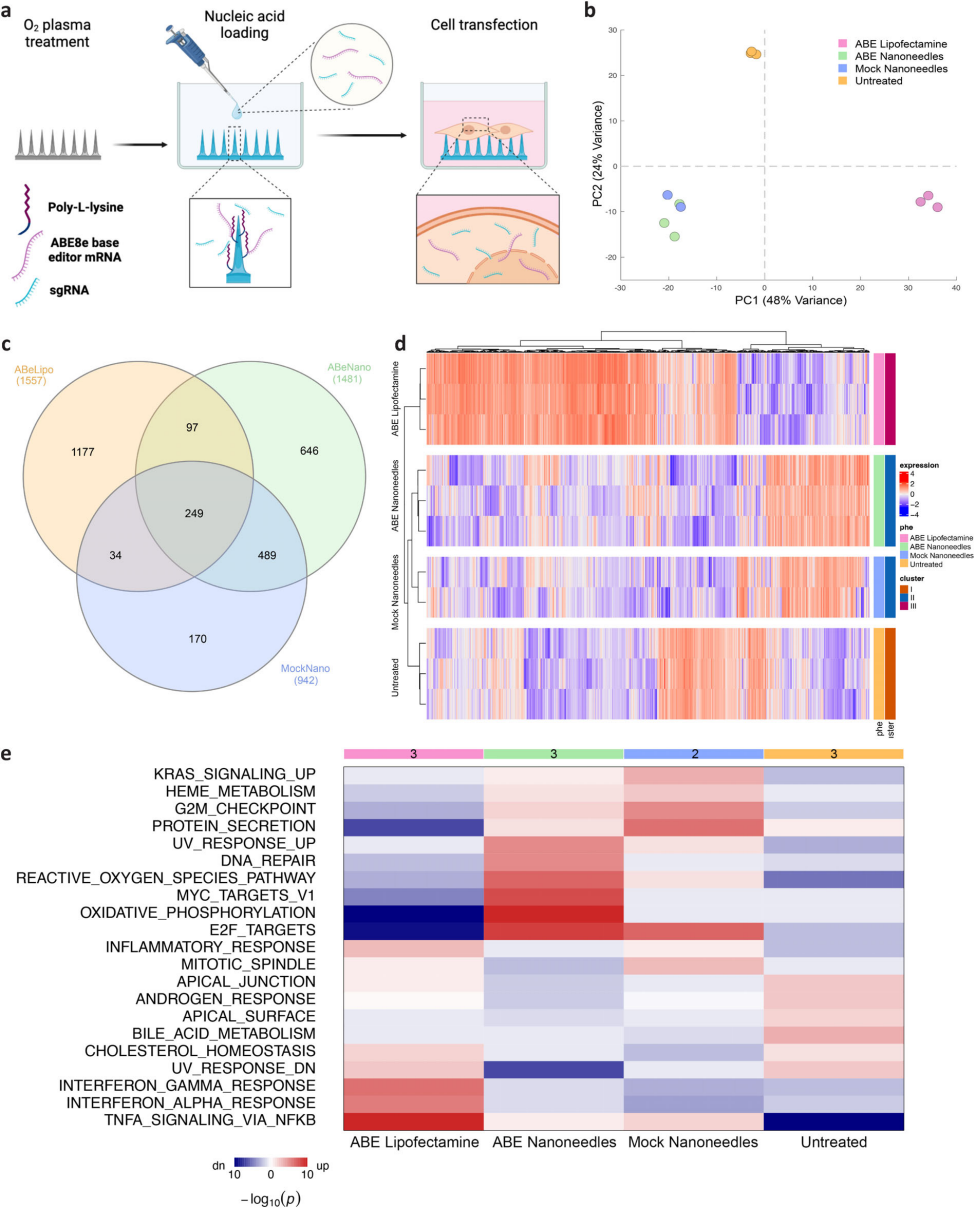
Nanoinjection of base editor has a minimal impact on gene expression in primary RDEB fibroblasts. a) Schematic diagram of the nanoinjection approach for intracellular base editor delivery. O_2_ plasma oxidized, and poly‐L‐lysine coated porous silicon nanoneedles are loaded with base editor mRNA and sgRNA targeting the variant of interest. Primary RDEB fibroblasts are then seeded on top of the nanoneedles. Upon interfacing, the nucleic acids are transfected across the plasma membrane into the cytoplasm where the mRNA can be translated into a base editor protein. Created in BioRender. b) Principal component analysis of gene expression for nanoinjected cells compared to untreated cells at 6 h post‐treatment. c) Venn diagram showing overlapping differentially expressed genes between different transfection conditions. d) Hierarchical cluster analysis and heatmap of differential gene expression profiles for the transfection conditions analyzed. e) Pathway enrichment analysis of the different transfection conditions. N ≥ 2 independent biological replicates.

We then loaded the mRNA coding for the ABE and the synthetic guide RNA (sgRNA) on nanoneedles treated with oxygen plasma and functionalized with poly‐L‐lysine (**Figure** **2**a). This treatment created a positively charged surface that enhanced the loading of negatively charged mRNAs onto the porous nanoneedles and facilitated nanoinjection. The porosity of the nanoneedles allowed controlled dissolution, ensuring the payload was released over the time required for cell spreading, thereby increasing the amount of payload effectively contributing to transfection.^[^ ^12^ ^]^ First, we assessed the impact of nanoinjection on the target cells’ phenotype, by comparing the transcriptome of untreated cells with those of cells 6 h following base‐editing nanoinjection (ABE nanoinjection), and cells nanoinjected using empty nanoneedles (mock nanoinjection). Cells 6 h following lipofection of the ABE base editor and sgRNA served as a benchmark. The principal component analysis (PCA) showed separation of the untreated and transfected cells into three distinct groups (Figure 2b). The first principal component (PC1) accounted for 48% of the variability, with the second principal component (PC2) accounting for 24%. The lipofection group was the most different from the untreated group, on account of its larger distance along PC1 and a comparable distance along PC2, with respect to the nanoinjection groups. The ABE and mock nanoinjection groups clustered together and differed from the untreated group mostly along PC2. The early timepoint chosen for this study may be the reason for the similarity between ABE and mock nanoinjection groups, as 6 h could only be capturing the start of translation and early onset of the impact of the base editor on the transcriptomic profile.^[^ ^35^ ^]^ Overall, lipofection, considered a mild transfection approach, induced larger transcriptomic changes than nanoinjection, confirming the minimally perturbative nature of nanoinjection.

The analysis of differential gene expression supported the indications from PCA, with lipofection regulating more genes (1557) than either nanoinjection condition (Figure 2c). The mock and ABE nanoinjection conditions showed notable similarity in gene expression patterns, sharing 738 common differentially expressed genes out of the 942 and 1481 regulated genes, respectively. However, the ABE nanoinjection group also affected 743 other genes not upregulated in the mock nanoinjection group, likely arising from the introduction of foreign RNA in the cell or the effects of the mRNA expression and base editing process. ABE nanoinjection and lipofectamine shared the regulation of 346 genes, likely related to the cellular response to the delivery of exogenous genetic material.

Cluster analysis provided further insights into the impact of the different treatments on gene expression patterns. The hierarchical clustering dendrogram and corresponding heatmap illustrated distinct separation among the four experimental groups, with replicates clustering closely together, indicating high reproducibility within each condition (Figure 2d). The analysis identified three primary clusters (untreated, nanoinjection and lipofection), reflecting the relative similarity between mock and ABE nanoinjection, consistent with the PCA analysis and differentially expressed gene overlap observed in the Venn diagram (Figure 2b,c). The analysis of cluster proximity suggested that mock nanoinjection induced the least transcriptomic changes out of all groups, preserving a gene expression profile most similar to the baseline of the untreated cells. The ABE nanoinjection formed the second closest cluster to untreated cells, indicating that ABE nanoinjection induces more transcriptomic changes compared to mock nanoinjection, likely due to the introduction of the gene‐editing RNAs. However, it is important to note that despite being further from the untreated group, the ABE nanoinjection samples are remarkably similar to the mock samples, suggesting that the changes are relatively modest. The ABE lipofectamine‐treated cells on the other hand, formed a separate cluster, furthest from the untreated group, underscoring more extensive transcriptomic modifications induced by lipofection. The clustering analysis corroborated the PCA, reinforcing the observation that nanoinjection yielded less alteration to the gene expression profiles than alternative transfection approaches (Figure 2d).

To assess the functional impact of the transcriptomic alterations, we performed pathway enrichment analysis, which highlighted known effects of nanoneedle interfacing with cells, and effects specific to the base editing process (Figure 2e). The upregulation of cell cycle‐related and signaling pathways such as E2F targets, G2 M checkpoint, and KRAS signaling likely reflected the cellular response to the increased rates of cell division and reduced motility induced by cells pinning on nanoneedles.^[^ ^31^, ^36^ ^]^ The increase in oxidative stress is a known feature of nanoneedle interfacing.^[^ ^37^ ^]^ Nanoinjection also displayed depletion of the cholesterol homeostasis, fatty acid metabolism, apical junction and apical surface pathways. These mechanisms are likely involved in the remodeling of the cell membrane occurring upon nanoneedle interfacing.^[^ ^38^, ^39^ ^]^ Only ABE nanoinjection yielded enrichment of the DNA repair mechanism, oxidative phosphorylation and MYC targets and UV response, likely indicating a response to the onset of the editing process. Indeed, base editing induces single‐strand DNA nicking and nucleotide deamination, which is repaired by non‐DSB DNA repair mechanisms such as base and alternative excision repair.^[^ ^40^ ^]^ This could explain the upregulation of the DNA damage response and DNA editing mechanisms, including the UV response pathway that comprises genes related to DNA damage.

Importantly nanoinjection downregulated inflammatory response, interferon signaling and weakly upregulated TNFA pathways, highlighting its non‐immunogenic nature.^[^ ^12^ ^]^ In contrast, these pathways were highly enriched in the ABE lipofectamine‐treated cells, supporting the immunogenicity of lipid‐based delivery and their potential to increase cell stress, reduce cell viability and induce apoptosis. Interestingly, there was a lack of cell cycle and DNA damage response following lipofection that might reflect a more efficient delivery by nanoinjection due to the direct access to the cytosol, yielding an earlier activation of the base editing processes.

These results together indicated that nanoinjection perturbed gene expression to a lesser extent than lipofection. The nanoneedle groups displayed fewer and more specific changes, with the ABE nanoinjection affecting specific pathways, likely due to the base editing process or RNA introduction. This highlighted the differential impact of delivery methods on gene expression, emphasizing the need to carefully consider the choice of delivery technique in genetic manipulation studies. Overall, these findings confirm the excellent safety profile of nanoinjection for gene delivery, supporting its suitability for the transfection of base editors.

### 2.3 Gene Correction by Nanoinjection of Adenine Base Editor

**Figure 3:**
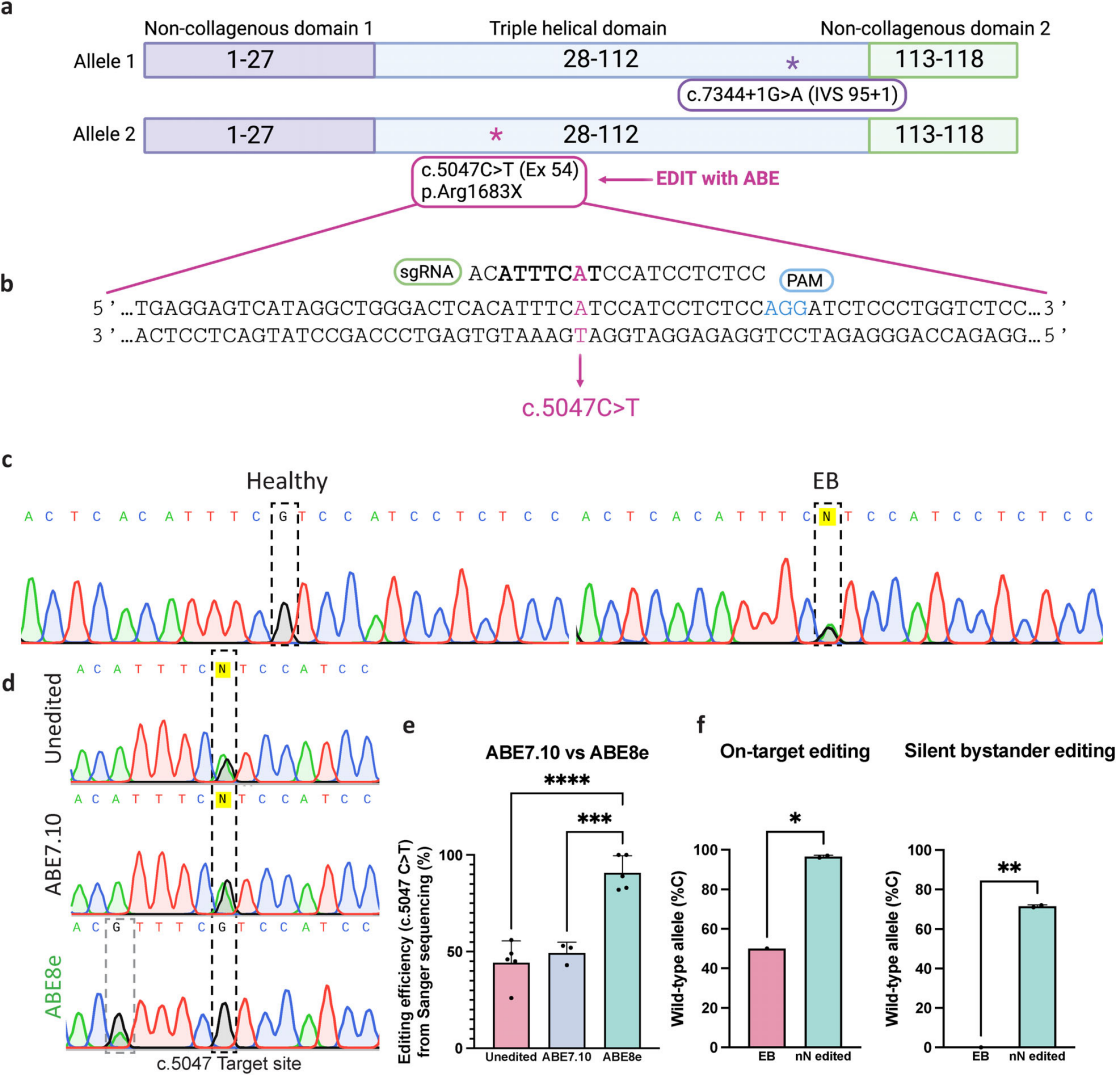
In vitro nanoinjection of adenine base editor leads to *COL7A1* gene correction. a) Schematic showing the compound heterozygous variant in primary RDEB fibroblasts. b) DNA sequence surrounding the RDEB c.5047 C>T variant. A chemically modified sgRNA used for the base editing experiments was designed to overlap the variant at position 8 of the 20 base pair protospacer (shown in pink). The editing window is shown in bold (position 3–9), and the PAM sequence required for the base editor binding is highlighted in blue. c) Sanger sequencing chromatograms showing the heterozygous RDEB variant compared to healthy donor DNA. d) Sanger sequencing chromatograms showing correction of the c.5047 G>A variant using ABE7.10 and ABE8e delivered using nanoinjection in primary RDEB fibroblasts. Dashed line surrounds the targeted base. Smaller grey dotted line box shows the bystander variant following editing with ABE8e. e) Quantification of editing efficiency for ABE7.10 (n =  3) and ABE8e (n = 5) compared to the unedited primary RDEB fibroblasts (n = 5). Data are presented as mean values ± S.D. Statistical significance was tested with one‐way ANOVA followed by Šidák's multiple comparison post‐hoc test. *: p < 0.05, **: p < 0.01, ***: p < 0.001, ****: p < 0.0001. f) Next generation sequencing analysis confirms on target editing in ABE8e nanoinjected cells (n = 2) compared to untreated (n = 1) (left) and shows the rate of bystander editing (right). Data are presented as mean values ± S.D. Statistical significance tested using an unpaired students t‐test. *: p < 0.05, **: p < 0.01; RDEB – recessive dystrophic epidermolysis bullosa.

Next, we examined nanoinjection efficiency and the ability of the base editor to correct target pathogenic variants in *COL7A1*. Our aim was to edit the recurrent RDEB c.5047 C>T pathogenic variant found in the triple helical domain (Exon 54) of *COL7A1*, which causes a premature termination codon (PTC). This variant converts the arginine at position 1683 into a stop codon (p.Arg1683Ter), resulting in a truncated mRNA transcript, suppressing the production of C7 protein (**Figure** **3**a). The c.5047 C>T variant is located at an appropriate distance from an “NGG” protospacer adjacent motif (PAM) and falls in the editing window of the base editor (position 8), making the reverse strand (which reflects a G>A variant) suitable for adenine base editing (Figure 3b). We characterised the heterozygous variant by Sanger sequencing of the region of interest and observed a double A/G peak in the primary RDEB fibroblasts compared to a single G peak in healthy fibroblasts (Figure 3c). In order to identify an efficient adenine base editing approach for nanoinjection, we delivered mRNA for the base editor ABE7.10 or ABE8e and sgRNA cargo by nanoinjection and assessed the editing efficiency. We observed that ABE8e achieved highly efficient correction of the RDEB variant in contrast to a negligible change using ABE7.10 (Figure 3d). The average C content achieved by nanoinjection using ABE8e was 90.8%, with two out of three replicates achieving 100% correction. The ABE8e editing was significantly more than ABE7.10 (49.3%) and the negative control of cells seeded on empty nanoneedles (44.4%) (Figure 3e). We validated the Sanger sequencing results using next generation sequencing (NGS), showing 96.5% C at the c.5047 locus in ABE8e nanoneedle edited cells compared to 50% in unedited cells. To account for the heterozygous nature of the variant, we calculated the correction efficiency and found it to be 93%, indicating that the majority of cells will possess the wild‐type G, thereby restoring Arg in the amino acid sequence (Figure 3f). The Sanger sequencing also revealed a double A/G peak at position c.5052 in ABE8e edited cells, which occurs due to bystander editing (Figure 3d; Figure S1a, Supporting Information), a phenomenon where an additional targetable nucleotide within, or near the editing window undergoes unintended editing.^[^ ^41^ ^]^ Bystander editing is a well‐documented limitation of the base editing system, and the observed levels of these edits reflect the enzyme's function rather than any effect of the nanoinjection process.^[^ ^10^, ^41^ ^]^ Although 71.5% of the transcripts contained the bystander edit as confirmed by NGS, the change of A>G at position 3 in the protospacer results in a silent variant (TT**A**>TT**G**), not affecting the asparagine in the amino acid sequence (Figure S1b, Supporting Information). These results demonstrate successful nanoneedle editing in primary RDEB fibroblasts in vitro and a superior editing efficiency of ABE8e over ABE7.10 leading to efficient correction of the c.5047 C>T variant.

### 2.4 No Detectable Off‐Target DNA and RNA Editing Following ABE in Primary RDEB Fibroblasts

**Figure 4:**
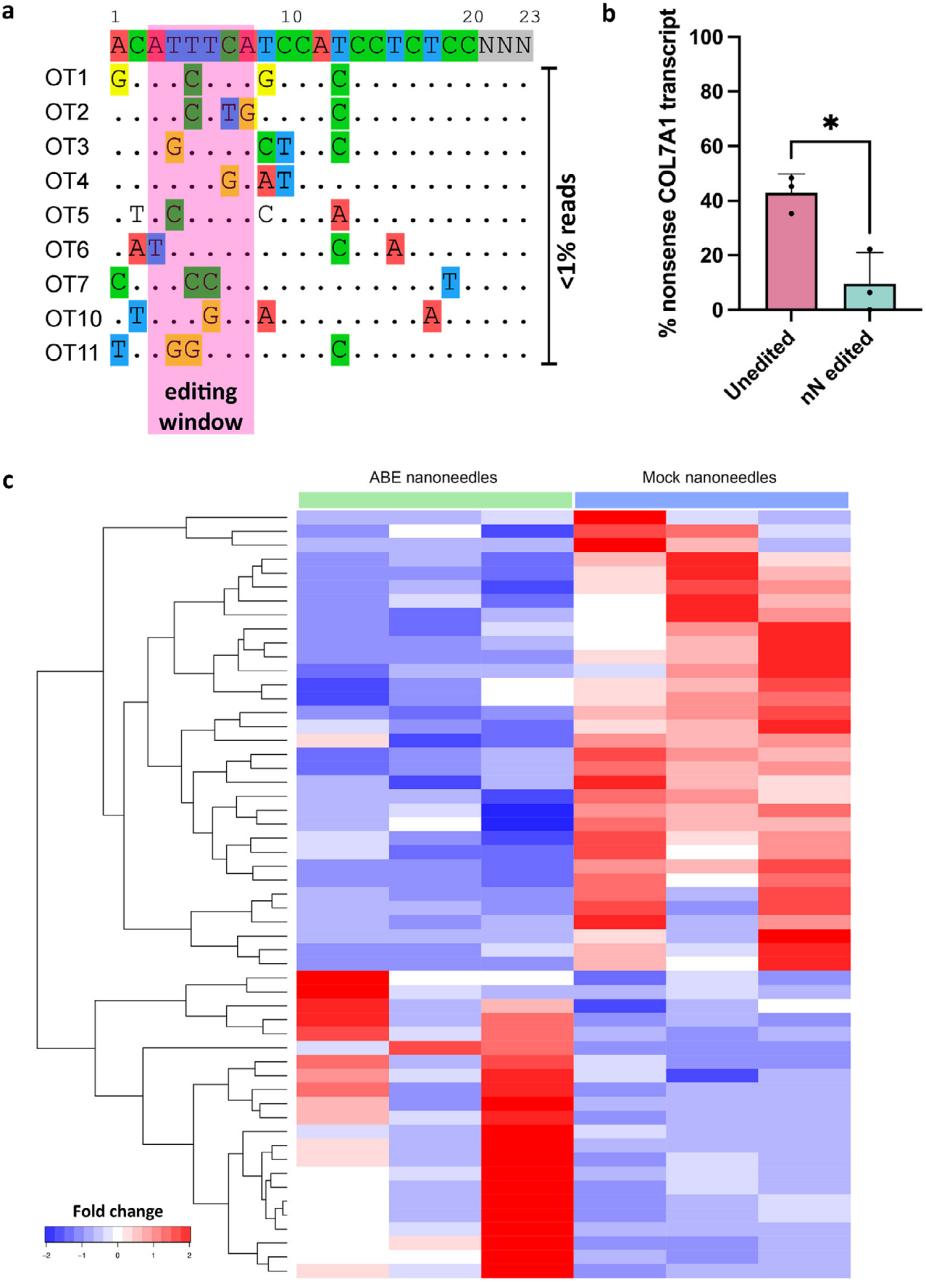
Assessment of changes in the genome and transcriptome following nanoneedle editing of primary RDEB fibroblasts. a) Next generation amplicon sequencing analysis of the top 9 out of 10 predicted off target sites in the genome showed no detectable edits above the 1% threshold within the editing window and surrounding bases. Mismatch bases between the sgRNA and potential off‐target binding sites are highlighted in different colours. b) Quantification of the amount of RNA transcripts containing the nonsense c.5047 T>C variant in the nanoneedle edited primary RDEB fibroblasts compared to unedited. Data are presented as mean values ± S.D. Statistical significance was tested using an unpaired students t‐test. *: p < 0.05. N = 3 independent biological samples. c) Heatmap of the top 55 differentially expressed genes (p < 0.01) in nanoneedle edited and unedited primary RDEB fibroblasts.

Having achieved highly efficient gene correction using nanoinjection, we next sought to assess the safety profile of the nanoneedle editing. Off‐target DNA and RNA editing activity of the ABE by deamination at undesired sites may be associated with oncogenic transformation or cytotoxicity, therefore posing a safety concern.^[^ ^42^ ^]^ We investigated the previously reported top 9 putative off‐target sites by NGS (Table S1, Supporting Information).^[^ ^10^ ^]^ Amplicon sequencing confirmed no relevant off‐target editing in the predicted editing window of the target sites (1% or less edited reads) (**Figure** **4**a), in line with reported negligible off‐target effects of ABE8e.^[^ ^43^ ^]^ Interestingly, when investigating the expected off‐target variants, we observed other single nucleotide DNA changes outside of the ABE editing window (Table S2, Supporting Information). There was no repetition of the variants across the different probes, suggesting that these single nucleotide variants occur due to mechanisms other than the base editing. A possible explanation may be an increased rate of endogenous APOBEC driven variants in RDEB cells, which has been reported to be higher in RDEB skin compared to healthy control.^[^ ^44^ ^]^ Additionally, variants other than ones attributed to APOBEC signature were also identified, suggesting influence from other mechanisms or random spontaneous variants occurring in RDEB cells. Of note, a study on cancer in RDEB reported that the DNA damage repair mechanism in RDEB squamous cell carcinoma (SCC) is active at a comparable level to wild‐type SCC; thus the higher burden of genetic alterations observed is likely a reflection of the increased rate of genetic changes and DNA damage from stress over time rather than impaired DNA repair.^[^ ^44^ ^]^

We also examined the transcriptomic changes in the edited cells 7–9 passages after editing, as an indicator of the long‐term effects of the gene correction and how this affects the cell phenotype and behavior.^[^ ^45^ ^]^ The non‐edited cells exhibited 42.9% expression of the mutant transcript. This aligns with the expected frequency of 50% mutated transcript in the heterozygous cells, of which we are likely observing a slight reduction due to nonsense‐mediated decay of the mutant mRNA. In the edited cells, the wild‐type *COL7A1* transcript was restored, with only 9.5% of the mutant transcript remaining (Figure 4b). The transcriptomic signature confirmed the observed genomic correction, and further indicates that it translates to gene expression and is successfully sustained over time. Analysis of transcriptome‐wide A‐to‐I conversion was carried out to assess the level of off‐target RNA editing.^[^ ^46^ ^]^ This found no remaining changes, most likely due to the transient nature of cellular RNAs, which is encouraging in terms of long‐term safety of base editing.

To gain a deeper understanding of the phenotypical effects following base editing, especially the impact of restoring wild‐type C7, we studied the differentially expressed genes between nanoneedle edited and untreated cells (Figure 4c; Figure S2, Table S3, Supporting Information). In the nanoneedle edited cells, we observed downregulation of genes associated with in cell–cell adhesion (*DSG1*) and ECM regulation (*ZNF469*), signifying changes in the ECM and cell structure dynamics, which could potentially be explained by an increase in C7 production. The downregulation of *MUC1*, which is involved in modulating cell membrane shape, particularly during interactions with cells or surfaces like nano‐patterns, can be likely attributed to the functional restoration of C7.^[^ ^47^ ^]^ This is evidenced by the fact that the cells were passaged on tissue culture plastic prior to the transcriptomic analysis, and this downregulation did not appear in nanoinjection controls without editing. Additionally, upregulation of *IGF2BP2* promotes cell proliferation by stabilizing m6A‐modified mRNAs like *MYC*, enhancing their translation, and protecting them from degradation, thereby sustaining the expression of key growth‐related genes.^[^ ^48^ ^]^ The increased expression of cell cycle regulators such as *DBF4B* and *HASPIN* in the edited primary RDEB fibroblasts further supports the notion of enhanced proliferation, suggesting that nanoneedle edited cells might be in a healthier, more robust state.^[^ ^49^ ^]^ Importantly, we observed that the pathways that were altered following nanoneedle interfacing (Figure 2e), returned to normal a few passages after treatment (Figure 4c), highlighting the transient nature of the nanoneedle effect on the phenotype as reported previously.^[^ ^50^ ^]^ Overall, these observations portray the transition from abnormally behaving RDEB cells to ones capable of producing full‐length C7, restoring regular cell function in terms of cell mobility, adhesion and ECM structuring many passages after the gene correction.

### 2.5 Improved C7 Expression Following Base Editing Using Nanoinjection

**Figure 5:**
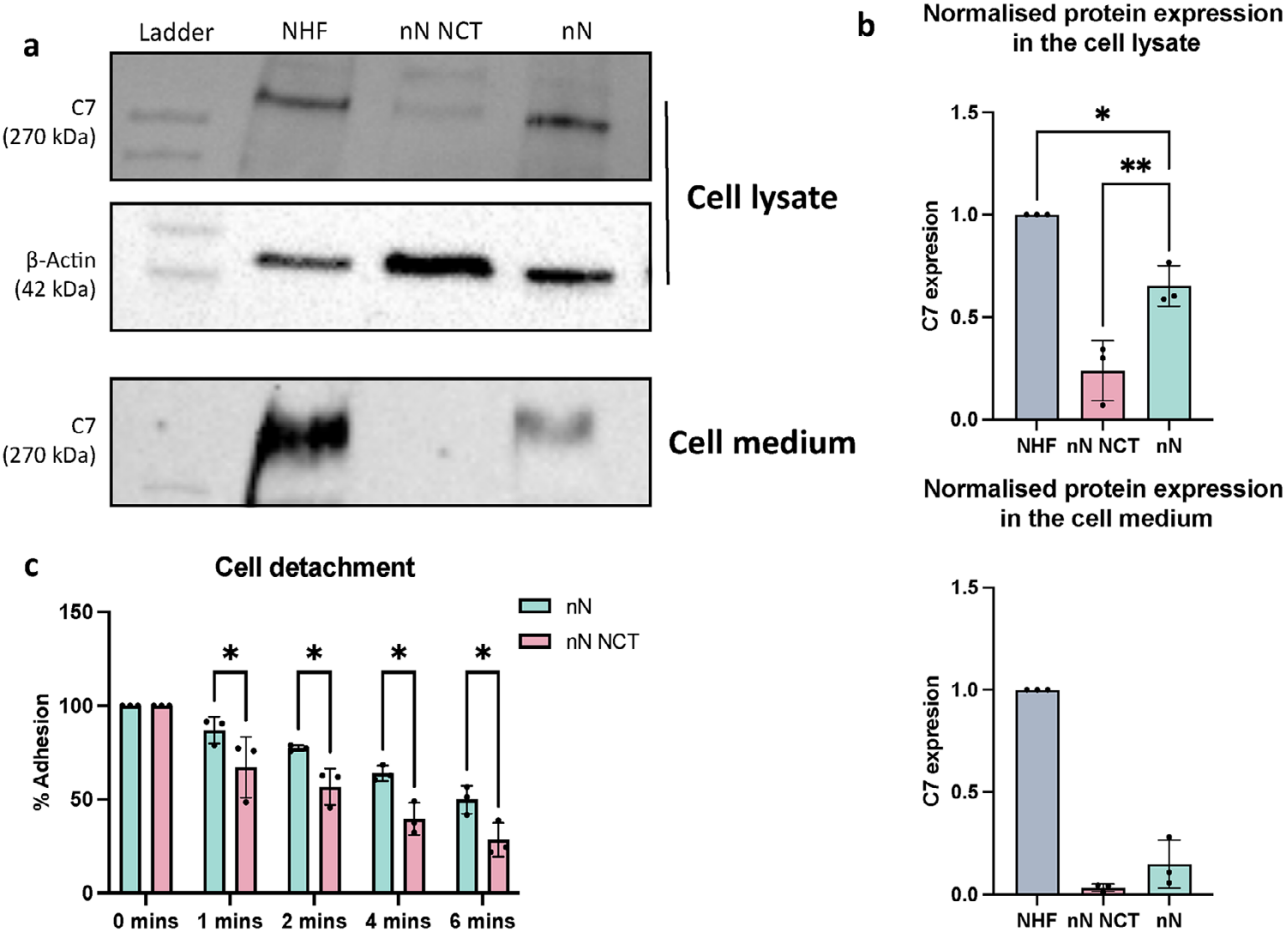
Nanoneedle editing of primary RDEB fibroblasts improves type VII collagen expression and cell adhesion. a) Type VII collagen (C7) western blots show an increase in the expression of full‐length C7 in the cell lysate and secreted into the medium for nanoneedle edited primary RDEB fibroblasts. NHF: primary normal human skin fibroblasts; nN NCT: nanoneedles negative control in the absence of the base editor system, nN: nanoneedle treatement with base editing. b) Densitometric quantification of normalized protein expression in the cell lysate and cell medium confirms the increased expression of C7 in the edited cells compared to the negative control. Data are presented as mean values ± S.D. Statistical significance was determined using one‐way ANOVA followed by Šidák's multiple comparison post‐hoc test. ns: non‐ significant, *: p < 0.05, **: p < 0.01, ***: p < 0.001, ****: p < 0.0001. N = 3 independent biological replicates. c) Trypsin detachment assay showing increased adhesion of edited cells. Data are presented as mean values ± S.D. Statistical significance was determined using multiple unpaired students t‐tests followed by two‐stage step‐up method of Benjamini, Krieger and Yekutieli false discovery rate post‐hoc test. *: p < 0.05. N = 3 independent biological samples.

Following base editing using nanoinjection, we sought to measure whether the correction was sufficient to recover the expression of full‐length C7 expression in the nanoneedle edited cells. Western blotting of the cell lysate and of the proteins secreted in the cell medium showed an increase of full‐length C7 in the ABE8e edited cells compared to the untreated ones (**Figure** **5**a; Figures S3–S5, Supporting Information). Densitometric analysis of the western blots showed higher levels of full‐length C7 protein in nanoneedle edited primary RDEB fibroblasts compared to mock nanoinjection control, respectively 63.5% and 24.6% of the healthy fibroblasts control, and of C7 secreted in the cell medium, respectively 15.1% and 3.2% (Figure 5b). C7 secretion into the cell medium indicates the functional, full‐length C7 being produced, which requires post‐translational modifications before assembling into anchoring fibrils for integration at the dermal‐epidermal junction. We controlled for equal loading of secreted protein using the Ponceau S total protein stain (Figure S5b, Supporting Information). The edited primary RDEB fibroblasts still express and secrete lower amounts of full‐length C7 in comparison to healthy fibroblasts, possibly due to the limited time for protein synthesis and secretion over the 48 h culturing period. The secretion of C7 is a complex, multistep process involving translation and several post‐translational modifications both within the cell and after secretion, such as homo‐trimerization, dimerization, and lateral assembly into anchoring fibrils. Additionally as the RDEB and normal human fibroblasts (NHF) cells originate from different individuals, inter‐individual variability may contribute to the reduced C7 expression observed in the RDEB edited fibroblasts. Notably, reduced C7 expression and secretion were also reported when editing these RDEB cells using electroporation,^[^ ^10^ ^]^ suggesting that this effect is independent of the nanoinjection process. Extending the culture period for cells post‐editing may provide further insights into their full potential for C7 production.

A trypsin detachment assay showed significantly increased adherence capability of nanoneedle edited cells compared to the unedited control (63.9% and 39.5% attachment, respectively at 4 min; 49.9% and 28.4% at 6 min). Increased resistance to trypsin is reflective of stronger cell adhesion,^[^ ^51^ ^]^ which is likely conferred by the increased C7 content in the nanoneedle edited cells (Figure 5c).

## 3 Conclusion

In this study, we demonstrated the safe and efficient nanoinjection of a base editing payload into primary RDEB fibroblasts, achieving up to 100% correction efficiency of a heterozygous pathogenic variant responsible for RDEB and successfully restoring C7 production. Primary RDEB fibroblasts cultured on nanoneedles exhibited effective interfacing, spreading, and elongation, with a maintained fibroblast phenotype. The nanoneedles showed high cytocompatibility, with no significant decrease in cell viability. Additionally, nanoinjection, whether with base editor‐loaded or empty nanoneedles, induced fewer transcriptomic perturbations compared to lipofection, confirming their non‐immunogenic nature.

Nanoneedle editing of the c.5047 C>T pathogenic variant using ABE8e^[^ ^43^ ^]^ achieved high editing efficiency. Sanger sequencing and NGS of the target locus confirmed efficient in vitro gene correction. Analysis revealed no significant off‐target effects at the top ten predicted sites, and long‐term transcriptomic studies showed sustained gene correction (91.5% wild type transcript expression multiple passages post editing). Additionally, the nanoneedle edited primary RDEB fibroblasts exhibited transcriptomic changes that suggested improvements in cellular adhesion and ECM remodeling. These RNA‐level changes correlated with increased C7 protein expression and enhanced cell adhesion, indicating a functional improvement following gene correction.

Overall, these findings demonstrate that nanoneedles can effectively deliver base editor mRNA into primary RDEB fibroblasts in vitro, with a superior safety profile. Compared to conventional transfection methods such as lipofection. Leveraging semiconductor manufacturing nanoneedles can be produced at scale at competitive costs, making them an attractive option for minimally‐perturbing gene editing of primary cells. The improvement of the RDEB phenotype in nanoneedle edited cells underscores the potential of nanoneedles to support the manufacturing processes in cell therapies for treating genetic diseases.

## 4 Experimental Section

### Nanoneedle Fabrication

Porous silicon nanoneedles (pSi nNs) were fabricated by initially depositing a 120–140 nm silicon nitride layer onto 0.01 Ω‐cm, boron‐doped p‐type, 100 mm silicon wafers (University Wafers), followed by photolithographically patterning a 600 nm diameter disk array with a 2 µm pitch. The wafers were dehydrated at 200 °C for 20 min before spin‐coating NR9‐250P photoresist (Futurrex), which was then pre‐baked at 70 °C for 180 s. A mask aligner (MA/BA6, K‐Suss) was used for exposure, followed by post‐baking at 100 °C for 60 s, and development in a 3:1 (v/v) RD6: deionized water solution for 12 s. Front‐end reactive ion etching (RIE, Oxford NGP 80) was performed using CHF_3_ plasma at 55 mTorr, 150 W, 50 sccm for 155 s, followed by a 10 min O_2_ plasma treatment. The substrate was cleaned in a 1:4 (v/v) mixture of 50% hydrofluoric acid (HF) and deionized water for 2 min, dried under nitrogen stream, and immersed in an aqueous solution of 0.02 m silver nitrate (AgNO_3_, Sigma–Aldrich) and 10% HF for 120 s. Nanopillars, 7 µm in height and 600 nm in diameter, were formed by etching in a mixture of 1% (v/v) hydrogen peroxide and 10% (v/v) HF in deionized water for 7 min 30 s, followed by immersion in Type TFA etchant for 10 min to strip the silver. The final step involved back‐end RIE in SF_6_ plasma at 100 mTorr, 300 W, 20 sccm for 240 s to form nanoneedles with a height of 1.2–1.5 µm. The substrate was then diced into 8×8 mm chips using a DISCO Dicing Saw (DAD3230) before use.

### Nanoinjection

8×8 mm nanoneedle chips were oxidized by O_2_ plasma (100 W, ZEPTO‐W6, Diener Electronic), placed in a 24‐well plate and functionalized by submerging in 0.1 mg mL^−1^ poly‐L‐lysine (PLL) (Sigma) for 1 h at room temperature (RT). Following PLL functionalization, the nanoneedle chips were washed three times with 1xPBS (Gibco). For nanoinjection of nucleic acids, the nanoneedles were loaded with 200 ng ABE (4 ng µL^−1^) and 400 ng sgRNA (8 ng µL^−1^) in 50 µL 1xPBS and incubated for 30 min. The c.5047 C>T variant in exon 54 of the *COL7A1* gene was targeted for correction using base editing as described in a recently published paper.^[^ ^10^ ^]^ 1×10^5^ fibroblasts were seeded on top of the nanoneedles. The cells were left to settle to the bottom of the well for 20 min before being transferred to the incubator and cultured for 48 h.

### Cell Culture

Primary fibroblasts isolated from a punch biopsy of a female RDEB patient were sourced from the cell bank at St. John's Institute of Dermatology, King's College London. The fibroblasts were cultured in Dulbecco's Modified Eagle Medium (DMEM) (Gibco) supplemented with 10% (v/v) Fetal Bovine Serum (FBS) (Gibco) and 1% (v/v) Penicillin/Streptomycin (Gibco) and grown under controlled conditions of 37 °C and 5% CO_2_. At ≈80% confluency, the cells were detached using TrypLE Express (Gibco), pelleted at 300 g for 5 min, resuspended in fresh media and seeded into new culture flasks. The MycoStrip kit (Invivogen) was used to confirm a negative mycoplasma status of the cell culture every two months.

### Flow Cytometry

Fibroblasts grown in a 24‐well plate on pSi nNs or flat silicon chips of the same size for 24 h. The following day, the chips were washed with 1xPBS, transferred to a fresh well, cells were harvested using TrypLE and centrifuged. The pellet was resuspended in 1xPBS/ 10% FBS with CellEvent Caspase‐3/7 (excitation 511/ emission 533) (Invitrogen) and incubated for 30 min at 37 °C. SYTOX AADvanced dye (546/647) (Invitrogen) was added in the last 5 min. After incubation the samples were kept on ice and measured with LSR Fortessa flow cytometer (BD Biosciences) equipped with 488 nm laser. The data was subsequently analyzed in FlowJo (BD Biosciences).

### DNA/RNA Isolation

Genomic DNA was isolated using the QIAamp DNA Mini Kit (Qiagen) and RNA was isolated using the RNeasy Kit (Qiagen), according to the manufacturer's instructions. The DNA and RNA were eluted in 30 µL AE buffer and 50 µL RNase‐free water, respectively.

The RNA extraction protocol was optimized for extraction from a small number of cells lysed directly on the nanoneedle chip. After the cells were seeded and left to incubate for 6 h, the nanoneedle chip was transferred to a fresh 24‐well, and the cells were lysed using 100 µL RLT buffer supplemented with 10% β‐mercaptoethanol, washed according to protocol and eluted in 10 µL RNase‐free water.

The NanoDrop 1000 Spectrophotometer (Thermo Scientific) was used to measure the concentration and purity of the nucleic acids at the A260/A280 and A260/A230 ratio, respectively. The RNA quantity and quality was also checked using Qubit RNA HS assay (Invitrogen) on a Qubit 3.0 fluorometer (Invitrogen).

### PCR Amplification

Regions of interest containing the targeted mutation and selected top 10 off‐target sites were amplified using polymerase chain reaction (PCR). A PCR master mix was prepared comprising of AmpliTaq Gold 360 DNA polymerase (Applied Biosystems), GC buffer (Applied Biosystems), forward and reverse primer at 10 mm concentration (Invitrogen and Sigma–Aldrich), nuclease‐free water (Invitrogen) and 10 ng of DNA starting material. The Veriti Thermo Cycler (Applied Biosystems) was used to amplify the DNA fragments. The amplification was verified using gel electrophoresis on 1.5% agarose gel with SYBR‐safe DNA dye (Invitrogen) and visualized on the UVP GelDoc‐It imaging system.

### Sanger Sequencing

The PCR product was purified from excess dNTPs and unincorporated primers using illustra ExoProStar (Cytiva) according to the manufacturer's protocol. Sanger sequencing was carried out by an external company (SourceBioscience), and the sequencing results were analyzed using SnapGene viewer (version 7.1) (Domantics)

### Next Generation Sequencing

High throughput (Next‐Generation Sequencing, NGS) targeted amplicon sequencing was performed to assess the on‐target editing efficiency and off‐target effects at previously reported putative sites.^[^ ^10^ ^]^

PCR products for the c.5047 target variant and off‐target sites (**Table** **1**) were prepared using primer sequences (Table S1, Supporting Information) with additional MiSeq NGS overhangs. The PCR products were purified using the MinElute PCR Purification Kit (Qiagen) and were subsequently sequenced on the MiSeq System (Illumina). Data quality control, genome alignment and quantification of single variant bases at sites of interest compared to the reference genome was performed as described in,^[^ ^10^ ^]^ to analyze the editing efficiency, bystander editing and generation of off‐target variants. A sample was excluded from the analysis due to poor sequencing quality.

**Table 1:** Top 9 off‐target sites.

Oligo name	Target seq 5′‐3′	Gene	Locus
1‐off	GCATCTCAGCCACCCTCTCCAGG	ARL10	NM_0 013 17948.2: 720.753
2‐off	ACATCTTGTCCACCCTCTCCAGG	RAB7B	NM_177 403.6: 2535.2557
3‐off	ACAGTTCACTCACCCTCTCCTGG	ANKFN1	NM_0 013 70326.1: 773.806
4‐off	ACATTTGAATCATCCTCTCCTGG	ADAMTS12	AC016613.7: 79 777.79799
5‐off	ATACTTCACCCAACCTCTCCTGG	SPOCK1	AC236725.3: 1115.1137
6‐off	AATTTTCATCCACCCACTCCTGG	GSAP intron	AC073635.8: 123 552.123574
7‐off	CCATCCCATCCATCCTCTTCTGG	AC009410.1‐AC074019.1 Intergenic	AC012070.8: 95 786.96169
10‐off	ATATTGCAACCATCCTCACCTGG	DNER Intron	AC007559.3: 31 111.31133
11‐off	TCAGGTCATCCACCCTCTCCAGG	RP5‐884C9.2‐LINC01343 Intergenic	AL139158.11: 91 786.91808

Forward primer NGS overhang **CGTCGGCAGCGTCAGATGTGTATAAGAGACAG**

Reverse primer NGS overhang **GTCTCGTGGGCTCGGAGATGTGTATAAGAGACAG**

### RNA Sequencing and Transcriptomic Analysis

RNA was sequenced externally (Novogene) and at the Hirszfeld Institute of Immunology and Experimental Therapy in Poland. RNA‐seq analyses were executed in R 4.2.2 and Bioconductor 3.16. In brief, fastq files were subjected to quality filtering using Rfastp Bioconductor package (version 1.10.0).^[^ ^52^ ^]^ Filtered reads were aligned with Rsubread Bioconductor package (version 2.14.2) to human reference genome GENCODE Release 43 (GRCh38.p13) to produce BAM files. Subsequently aligned reads were summarized to read counts (raw counts) using Rsubread Bioconductor package and GENCODE Release 43 (GRCh38.p13) gene annotation.^[^ ^53^, ^54^ ^]^ Lowly expressed genes were discarded by using filterByExpr() function in the edgeR package (version 3.42.4).^[^ ^55^ ^]^ Next the data was transformed using the trimmed mean of the M value method. Subsequently, the quasi‐likelihood F test was used for the differential analysis. Differentially expressed genes were identified with |logFC| ≥ 1 and false discovery rate (FDR) ≤ 0.05 thresholds. Gene set enrichment was performed with the CAMERA algorithm implemented in the CMScaller R package.^[^ ^56^ ^]^ The hallmark gene sets (h.all.v2022.1.Hs.symbols) provided by the Molecular Signatures Database were used to identify deregulated pathways.^[^ ^57^ ^]^ PCA plots were generated in tidyverse ecosystem, whereas heatmap was generated in the ComplexHeatmap package.^[^ ^58^ ^]^ Heatmaps were created using the clustermap function in Python's seaborn library, employing hierarchical clustering from the scipy library. REDItools v1.3 was utilized to quantify the percentage of A to I changes.^[^ ^59^ ^]^ A sample was excluded from the analysis due to poor sequencing quality.

### Protein Extraction and Western Blotting

Fibroblasts grown to 80% confluency in a 6‐well plate in DMEM (10% FBS, 1% P/S) were washed three times with 1xPBS and cultured for further 48 h in Opti‐MEM (Gibco), a serum‐free medium, supplemented with 20 ng/mL TGFβ‐2 (1% v/v) and 50 µg mL^−1^ ascorbic acid (5% v/v) to stimulate C7 production. For analysis of the secreted C7, the media was collected, and cleared of cell debris by centrifuging at 500 rotations per minute (rpm) for 5 min at 4 °C. The supernatant proteins were precipitated in 1:4 ratio with ice‐cold acetone overnight, collected by ultracentrifugation (18 000 g, 10 min) and resuspended in a urea Tris‐HCL buffer (Urea 9.5 m (Sigma), Tris‐HCL 1 m (Sigma), pH 8 and Water) by gentle pipetting. For analysis of intracellular proteins, the cells were rinsed in 1xPBS and lysed on ice with 100 µL of RIPA lysis buffer (Sigma) with 1% Proteinase Inhibitor Cocktail (Calbiochem). The lysate was stored at −80 °C overnight and centrifuged at 8000 g, 4 °C for 10 min the following day to remove cell debris.

The Pierce BCA Protein Assay Kit (Thermo Scientific) was used to quantify the total protein content in each sample and normalise the sample load for sodium dodecyl sulphate polyacrylamide gel electrophoresis (SDS‐PAGE) according to manufacturer's instructions.

The samples were prepared with 4x Sample buffer (Bio‐Rad) containing 10% β‐mercaptoethanol at 3:1 ratio and boiled at 95 °C for 10 min. The proteins were separated on a 4–20% SDS polyacrylamide gel (Bio‐Rad) at 100 V for 15 min, then 80 V for 70–80 min along with Spectra Multicolor High Range Protein Ladder (Thermo Scientific) to estimate the size of protein bands. The Turbo Transfer system (Bio‐Rad) was used to transfer the proteins from the gel to a PVDF membrane at 25 V for 10 min. Reversible Ponceau staining (Cell Signal) was used immediately after transfer to visualize the total proteins.

The blots were blocked with 5% milk PBS‐T for 1 h at RT. Subsequently they were probed for C7 overnight at 4 °C using a polyclonal rabbit anti‐Collagen VII primary antibody (Bio‐Rad, VPA00854) (1:1000) or β‐actin for 1 h at RT using anti‐β‐actin primary antibody (Santa‐Cruz Biotechnologies, sc‐47778) (1:2000). The respective secondary antibodies (goat anti‐rabbit horseradish‐peroxidase, HRP (Dako, P0448) and goat anti‐mouse HRP (Dako, P0447)) were incubated for at 1:2000 for 1 h at RT. Prior to imaging on the iBright FL1500 imaging system, the membranes were developed for 2 min in the dark using the SuperSignal West Femto Maximum Sensitivity Substrate (Thermo Scientific) kit for C7 and the ECL Prime Western Blotting Detection Reagent (Cytiva) for β‐actin. The bands were quantified using iBright analysis software and normalized against β‐actin.

### Trypsin Detachment Assay

Fibroblasts were seeded in a 96‐well plate at 1.5×10^4^ cells per well. The following day, cells were washed with 1XPBS and subsequently treated with trypsin/EDTA (Gibco) for 6, 4, 2, 1, and 0 min. Following incubation, the cells were washed twice with 1xPBS to remove detached cells. Adherent cells in the plate were stained with 0.5% crystal‐violet (Sigma) in distilled water for 30 min, lysed with 1% SDS (Sigma). The percentage of adherent cells was determined by the measure of the absorbance at 590 nm using a spectrophotometer.

## Conflict of Interest

The authors declare no conflict of interest.

## Author Contributions

S.A.M. and M.D. contributed equally to this work. S.A.M. performed methodology, investigation, conceptualization, formal analysis, and project administration. M.D. performed methodology, investigation, formal analysis, project administration, and wrote the original draft. C.W., C.G., N.S., K.R., P.K., and L.L. performed methodology, investigation, and formal analysis. L.L. performed methodology, formal analysis, acquired resources, and reviewed and edited the final manuscript. J.A.M. performed conceptualization, funding acquisition, acquired resources, and reviewed and edited the final manuscript. J.J.M. performed conceptualization, acquired resources, and wrote the original draft. C.C. performed conceptualization, methodology, formal analysis, resources, wrote the original draft, supervision, project administration, and funding acquisition.

## Supporting information



Supporting Information

## Data Availability

The data that support the findings of this study are available from the corresponding author upon reasonable request.

## References

[1] A. Bardhan , L. Bruckner‐Tuderman , I. L. C. Chapple , J.‐D. Fine , N. Harper , C. Has , T. M. Magin , M. P. Marinkovich , J. F. Marshall , J. A. McGrath , J. E. Mellerio , R. Polson , A. H. Heagerty , Nat. Rev. Dis. Primer 2020, 6, 78. 10.1038/s41572-020-0210-0 32973163

[2] C. Has , J. W. Bauer , C. Bodemer , M. C. Bolling , L. Bruckner‐Tuderman , A. Diem , J.‐D. Fine , A. Heagerty , A. Hovnanian , M. P. Marinkovich , A. E. Martinez , J. A. McGrath , C. Moss , D. F. Murrell , F. Palisson , A. Schwieger‐Briel , E. Sprecher , K. Tamai , J. Uitto , D. T. Woodley , G. Zambruno , J. E. Mellerio , Br. J. Dermatol. 2020, 183, 614. 32017015 10.1111/bjd.18921

[3] J. A. McGrath , A. Ishida‐Yamamoto , A. O'Grady , I. M. Leigh , R. A. Eady , J. Invest. Dermatol. 1993, 100, 366. 8454899 10.1111/1523-1747.ep12471830

[4] B. Brodsky , G. Thiagarajan , B. Madhan , K. Kar , Biopolymers 2008, 89, 345. 18275087 10.1002/bip.20958

[5] S. V. Guide , M. E. Gonzalez , I. S. Bağcı , B. Agostini , H. Chen , G. Feeney , M. Steimer , B. Kapadia , K. Sridhar , L. Q. Sanchez , F. Gonzalez , M. V. Ligten , T. J. Parry , S. Chitra , L. A. Kammerman , S. Krishnan , M. P. Marinkovich , N. Engl. J. Med. 2022, 387, 2211. 36516090 10.1056/NEJMoa2206663

[6] Z. Siprashvili , N. T. Nguyen , E. S. Gorell , K. Loutit , P. Khuu , L. K. Furukawa , H. P. Lorenz , T. H. Leung , D. R. Keene , K. E. Rieger , P. Khavari , A. T. Lane , J. Y. Tang , M. P. Marinkovich , JAMA 2016, 316, 1808. 27802546 10.1001/jama.2016.15588

[7] S. M. Lwin , F. Syed , W.‐L. Di , T. Kadiyirire , L. Liu , A. Guy , A. Petrova , A. Abdul‐Wahab , F. Reid , R. Phillips , M. Elstad , C. Georgiadis , S. Aristodemou , P. A. Lovell , J. R. McMillan , J. Mee , S. Miskinyte , M. Titeux , L. Ozoemena , R. Pramanik , S. Serrano , R. Rowles , C. Maurin , E. Orrin , M. Martinez‐Queipo , E. Rashidghamat , C. Tziotzios , A. Onoufriadis , M. Chen , L. Chan , et al., JCI Insight 2019, 4, e126243. 31167965 10.1172/jci.insight.126243 PMC6629162

[8] M. Arbab , Z. Matuszek , K. M. Kray , A. Du , G. A. Newby , A. J. Blatnik , A. Raguram , M. F. Richter , K. T. Zhao , J. M. Levy , M. W. Shen , W. D. Arnold , D. Wang , J. Xie , G. Gao , A. H. M. Burghes , D. R. Liu , Science 2023, 380, eadg6518. 36996170 10.1126/science.adg6518 PMC10270003

[9] G. A. Newby , J. S. Yen , K. J. Woodard , T. Mayuranathan , C. R. Lazzarotto , Y. Li , H. Sheppard‐Tillman , S. N. Porter , Y. Yao , K. Mayberry , K. A. Everette , Y. Jang , C. J. Podracky , E. Thaman , C. Lechauve , A. Sharma , J. M. Henderson , M. F. Richter , K. T. Zhao , S. M. Miller , T. Wang , L. W. Koblan , A. P. McCaffrey , J. F. Tisdale , T. A. Kalfa , S. M. Pruett‐Miller , S. Q. Tsai , M. J. Weiss , D. R. Liu , Nature 2021, 595, 295. 34079130 10.1038/s41586-021-03609-w PMC8266759

[10] A. Sheriff , I. Guri , P. Zebrowska , V. Llopis‐Hernandez , I. R. Brooks , S. Tekkela , K. Subramaniam , R. Gebrezgabher , G. Naso , A. Petrova , K. Balon , A. Onoufriadis , D. Kujawa , M. Kotulska , G. Newby , Ł. Łaczmański , D. R. Liu , J. A. McGrath , J. Jacków , Sci. Rep. 2022, 12, 19643. 36385635 10.1038/s41598-022-24184-8 PMC9666996

[11] M. Kucharski , P. Mrowiec , E. Ocłoń , Biotechnol. Prog. 2021, 37, e3152. 33774920 10.1002/btpr.3152

[12] C. Chiappini , E. De Rosa , J. O. Martinez , X. Liu , J. Steele , M. M. Stevens , E. Tasciotti , Nat. Mater. 2015, 14, 532. 25822693 10.1038/nmat4249 PMC4538992

[13] C. Chiappini , X. Liu , J. R. Fakhoury , M. Ferrari , Adv. Funct. Mater. 2010, 20, 2231. 21057669 10.1002/adfm.201000360 PMC2971684

[14] C. Chiappini , E. Tasciotti , J. R. Fakhoury , D. Fine , L. Pullan , Y.‐C. Wang , L. Fu , X. Liu , M. Ferrari , ChemPhysChem 2010, 11, 1029. 20162656 10.1002/cphc.200900914 PMC2920042

[15] F. Cunin , T. A. Schmedake , J. R. Link , Y. Y. Li , J. Koh , S. N. Bhatia , M. J. Sailor , Nat. Mater. 2002, 1, 39. 12618846 10.1038/nmat702

[16] M. A. Tölli , M. P. A. Ferreira , S. M. Kinnunen , J. Rysä , E. M. Mäkilä , Z. Szabó , R. E. Serpi , P. J. Ohukainen , M. J. Välimäki , A. M. R. Correia , J. J. Salonen , J. T. Hirvonen , H. J. Ruskoaho , H. A. Santos , Biomaterials 2014, 35, 8394. 24985734 10.1016/j.biomaterials.2014.05.078

[17] S. H. C. Anderson , H. Elliott , D. J. Wallis , L. T. Canham , J. J. Powell , Phys. Status Solidi A 2003, 197, 331.

[18] M. Kaasalainen , R. Zhang , P. Vashisth , A. A. Birjandi , M. S'Ari , D. A. Martella , M. Isaacs , E. Mäkilä , C. Wang , E. Moldenhauer , P. Clarke , A. Pinna , X. Zhang , S. A. Mustfa , V. Caprettini , A. P. Morrell , E. Gentleman , D. S. Brauer , O. Addison , X. Zhang , M. Bergholt , K. Al‐Jamal , A. A. Volponi , J. Salonen , N. Hondow , P. Sharpe , C. Chiappini , Nat. Commun. 2024, 15, 487. 38216556 10.1038/s41467-023-44581-5 PMC10786831

[19] T. Tanaka , L. S. Mangala , P. E. Vivas‐Mejia , R. Nieves‐Alicea , A. P. Mann , E. Mora , H.‐D. Han , M. M. K. Shahzad , X. Liu , R. Bhavane , J. Gu , J. R. Fakhoury , C. Chiappini , C. Lu , K. Matsuo , B. Godin , R. L. Stone , A. M. Nick , G. Lopez‐Berestein , A. K. Sood , M. Ferrari , Cancer Res. 2010, 70, 3687. 20430760 10.1158/0008-5472.CAN-09-3931 PMC3202607

[20] C. Chiappini , J. O. Martinez , E. De Rosa , C. S. Almeida , E. Tasciotti , M. M. Stevens , ACS Nano 2015, 9, 5500. 25858596 10.1021/acsnano.5b01490 PMC4733661

[21] E. J. Anglin , L. Cheng , W. R. Freeman , M. J. Sailor , Adv. Drug Delivery Rev. 2008, 60, 1266. 10.1016/j.addr.2008.03.017 PMC2710886 18508154

[22] T. Tieu , M. Alba , R. Elnathan , A. Cifuentes‐Rius , N. H. Voelcker , Adv. Ther. 2019, 2, 1800095.

[23] J. Salonen , E. Mäkilä , Adv. Mater. 2018, 30, 1703819. 10.1002/adma.201703819 29484727

[24] R. Elnathan , M. G. Barbato , X. Guo , A. Mariano , Z. Wang , F. Santoro , P. Shi , N. H. Voelcker , X. Xie , J. L. Young , Y. Zhao , W. Zhao , C. Chiappini , Nat. Rev. Mater. 2022, 7, 953.

[25] C. Chiappini , Y. Chen , S. Aslanoglou , A. Mariano , V. Mollo , H. Mu , E. De Rosa , G. He , E. Tasciotti , X. Xie , F. Santoro , W. Zhao , N. H. Voelcker , R. Elnathan , Nat. Protoc. 2021, 16, 4539. 34426708 10.1038/s41596-021-00600-7

[26] Y. Cao , M. Hjort , H. Chen , F. Birey , S. A. Leal‐Ortiz , C. M. Han , J. G. Santiago , S. P. Paşca , J. C. Wu , N. A. Melosh , Proc. Natl. Acad. Sci. USA 2017, 114, E1866. 28223521 10.1073/pnas.1615375114 PMC5347600

[27] H. Kim , C. Gu , S. A. Mustfa , D. A. Martella , C. Wang , Y. Wang , C. Chiappini , ACS Appl. Mater. Interfaces 2023, 15, 49964. 37769296 10.1021/acsami.3c07918 PMC10623508

[28] Z. Wang , L. Qi , Y. Yang , M. Lu , K. Xie , X. Zhao , E. H. C. Cheung , Y. Wang , X. Jiang , W. Zhang , L. Huang , X. Wang , P. Shi , Sci. Adv. 2020, 6, eaba4971. 32577522 10.1126/sciadv.aba4971 PMC7286670

[29] R. Elnathan , A. W. Holle , J. Young , M. A. George , O. Heifler , A. Goychuk , E. Frey , R. Kemkemer , J. P. Spatz , A. Kosloff , F. Patolsky , N. H. Voelcker , J. Nanobiotechnol. 2021, 19, 51. 10.1186/s12951-021-00795-7 PMC7890818 33596905

[30] H. Kim , H. Jang , B. Kim , M. K. Kim , D. S. Wie , H. S. Lee , D. R. Kim , C. H. Lee , Sci. Adv. 2018, 4, eaau6972. 30430139 10.1126/sciadv.aau6972 PMC6226283

[31] C. Wang , C. Gu , C. Popp , P. Vashisth , S. A. Mustfa , D. A. Martella , C. Spiteri , S. McLennan , N. Sun , M. Riddle , C. R. Eide , M. Parsons , J. Tolar , J. A. McGrath , C. Chiappini , ACS Nano 2024, 18, 14938. 38726598 10.1021/acsnano.4c00206 PMC11171749

[32] A. R. K. Kumar , J. Low , J. Lim , B. Myint , X. Sun , L. Wu , H. S. Cheng , S. Yip , C. Z. Ming Cheng , T. Manoharan , Y. J. Quek , Y. Shou , J. S. Tian , Y. Y. Ng , N. R. J. Gascoigne , N. S. Tan , R. Sugimura , G. Chia , A. M. Sze Cheung , M. Yawata , A. Tay , Biomaterials 2025, 317, 123079. 39842078 10.1016/j.biomaterials.2024.123079

[33] E. Maurizi , D. A. Martella , D. Schiroli , A. Merra , S. A. Mustfa , G. Pellegrini , C. Macaluso , C. Chiappini , Adv. Sci. Weinh. Baden‐Wurtt. Ger. 2022, 9, 2203257. 10.1002/advs.202203257 PMC9685449 36253148

[34] Y. Chen , S. Aslanoglou , T. Murayama , G. Gervinskas , L. I. Fitzgerald , S. Sriram , J. Tian , A. P. R. Johnston , Y. Morikawa , K. Suu , R. Elnathan , N. H. Voelcker , Adv. Mater. 2020, 32, 2000036. 10.1002/adma.202000036 32378244

[35] T. Jiang , J. M. Henderson , K. Coote , Y. Cheng , H. C. Valley , X.‐O. Zhang , Q. Wang , L. H. Rhym , Y. Cao , G. A. Newby , H. Bihler , M. Mense , Z. Weng , D. G. Anderson , A. P. McCaffrey , D. R. Liu , W. Xue , Nat. Commun. 2020, 11, 1979. 32332735 10.1038/s41467-020-15892-8 PMC7181807

[36] H. Persson , Z. Li , J. O. Tegenfeldt , S. Oredsson , C. N. Prinz , Sci. Rep. 2015, 5, 18535. 26691936 10.1038/srep18535 PMC4686997

[37] X. Zhu , M. F. Yuen , L. Yan , Z. Zhang , F. Ai , Y. Yang , P. K. N. Yu , G. Zhu , W. Zhang , X. Chen , Adv. Healthcare Mater. 2016, 5, 1157. 10.1002/adhm.201670048 27226035

[38] S. Gopal , C. Chiappini , J. Penders , V. Leonardo , H. Seong , S. Rothery , Y. Korchev , A. Shevchuk , M. M. Stevens , Adv. Mater. 2019, 31, 1806788. 10.1002/adma.201806788 PMC6606440 30680803

[39] G. Lou , G. Anderluzzi , S. T. Schmidt , S. Woods , S. Gallorini , M. Brazzoli , F. Giusti , I. Ferlenghi , R. N. Johnson , C. W. Roberts , D. T. O'Hagan , B. C. Baudner , Y. Perrie , J. Controlled Release 2020, 325, 370. 10.1016/j.jconrel.2020.06.027 32619745

[40] S. Gu , Z. Bodai , Q. T. Cowan , A. C. Komor , Gene Genome Ed. 2021, 1, 100005. 34368792 10.1016/j.ggedit.2021.100005 PMC8341163

[41] H. A. Rees , D. R. Liu , Nat. Rev. Genet. 2018, 19, 770. 30323312 10.1038/s41576-018-0059-1 PMC6535181

[42] N. M. Gaudelli , A. C. Komor , H. A. Rees , M. S. Packer , A. H. Badran , D. I. Bryson , D. R. Liu , Nature 2017, 551, 464. 29160308 10.1038/nature24644 PMC5726555

[43] N. M. Gaudelli , D. K. Lam , H. A. Rees , N. M. Solá‐Esteves , L. A. Barrera , D. A. Born , A. Edwards , J. M. Gehrke , S.‐J. Lee , A. J. Liquori , R. Murray , M. S. Packer , C. Rinaldi , I. M. Slaymaker , J. Yen , L. E. Young , G. Ciaramella , Nat. Biotechnol. 2020, 38, 892. 32284586 10.1038/s41587-020-0491-6

[44] R. J. Cho , L. B. Alexandrov , N. Y. den Breems , V. S. Atanasova , M. Farshchian , E. Purdom , T. N. Nguyen , C. Coarfa , K. Rajapakshe , M. Prisco , J. Sahu , P. Tassone , E. J. Greenawalt , E. A. Collisson , W. Wu , H. Yao , X. Su , C. Guttmann‐Gruber , J. P. Hofbauer , R. Hashmi , I. Fuentes , S. C. Benz , J. Golovato , E. A. Ehli , C. M. Davis , G. E. Davies , K. R. Covington , D. F. Murrell , J. C. Salas‐Alanis , F. Palisson , et al., Sci. Transl. Med. 2018, 10, eaas9668. 30135250 10.1126/scitranslmed.aas9668

[45] G. E. McAuley , G. Yiu , P. C. Chang , G. A. Newby , B. Campo‐Fernandez , S. T. Fitz‐Gibbon , X. Wu , S.‐H. L. Kang , A. Garibay , J. Butler , V. Christian , R. L. Wong , K. A. Everette , A. Azzun , H. Gelfer , C. S. Seet , A. Narendran , L. Murguia‐Favela , Z. Romero , N. Wright , D. R. Liu , G. M. Crooks , D. B. Kohn , Cell 2023, 186, 1398. 36944331 10.1016/j.cell.2023.02.027 PMC10876291

[46] H. A. Rees , C. Wilson , J. L. Doman , D. R. Liu , Sci. Adv. 2019, 5, eaax5717. 31086823 10.1126/sciadv.aax5717 PMC6506237

[47] C.‐H. Lu , K. Pedram , C.‐T. Tsai , T. Jones , X. Li , M. L. Nakamoto , C. R. Bertozzi , B. Cui , Nat. Commun. 2022, 13, 3093. 35654773 10.1038/s41467-022-30610-2 PMC9163104

[48] H. Huang , H. Weng , W. Sun , X. Qin , H. Shi , H. Wu , B. S. Zhao , A. Mesquita , C. Liu , C. L. Yuan , Y.‐C. Hu , S. Hüttelmaier , J. R. Skibbe , R. Su , X. Deng , L. Dong , M. Sun , C. Li , S. Nachtergaele , Y. Wang , C. Hu , K. Ferchen , K. D. Greis , X. Jiang , M. Wei , L. Qu , J.‐L. Guan , C. He , J. Yang , J. Chen , Nat. Cell Biol. 2018, 20, 285. 29476152 10.1038/s41556-018-0045-z PMC5826585

[49] K. Engeland , Cell Death Differ. 2018, 25, 114. 29125603 10.1038/cdd.2017.172 PMC5729532

[50] C. S. Hansel , S. W. Crowder , S. Cooper , S. Gopal , M. João Pardelha da Cruz , L. de Oliveira Martins , D. Keller , S. Rothery , M. Becce , A. E. G. Cass , C. Bakal , C. Chiappini , M. M. Stevens , ACS Nano 2019, 13, 2913. 30829469 10.1021/acsnano.8b06998 PMC6439438

[51] S.‐A. Hong , S.‐E. Kim , A.‐Y. Lee , G.‐H. Hwang , J. H. Kim , H. Iwata , S.‐C. Kim , S. Bae , S. E. Lee , Mol. Ther. J. Am. Soc. Gene Ther. 2022, 30, 2664. 10.1016/j.ymthe.2022.06.005 PMC9372317 35690907

[52] W. Wang , J.‐D. Luo , T. Carroll , 2024.

[53] A. Frankish , M. Diekhans , I. Jungreis , J. Lagarde , J. E. Loveland , J. M. Mudge , C. Sisu , J. C. Wright , J. Armstrong , I. Barnes , A. Berry , A. Bignell , C. Boix , S. Carbonell Sala , F. Cunningham , T. Di Domenico , S. Donaldson , I. T. Fiddes , C. García Girón , J. M. Gonzalez , T. Grego , M. Hardy , T. Hourlier , K. L. Howe , T. Hunt , O. G. Izuogu , R. Johnson , F. J. Martin , L. Martínez , S. Mohanan , et al., Nucleic Acids Res. 2021, 49, D916. 33270111 10.1093/nar/gkaa1087 PMC7778937

[54] Y. Liao , G. K. Smyth , W. Shi , Nucleic Acids Res. 2019, 47, e47. 30783653 10.1093/nar/gkz114 PMC6486549

[55] M. D. Robinson , D. J. McCarthy , G. K. Smyth , Bioinforma. Oxf. Engl. 2010, 26, 139. 10.1093/bioinformatics/btp616 PMC2796818 19910308

[56] D. Wu , G. K. Smyth , Nucleic Acids Res. 2012, 40, e133. 22638577 10.1093/nar/gks461 PMC3458527

[57] A. Liberzon , C. Birger , H. Thorvaldsdóttir , M. Ghandi , J. P. Mesirov , P. Tamayo , Cell Syst. 2015, 1, 417. 26771021 10.1016/j.cels.2015.12.004 PMC4707969

[58] Z. Gu , R. Eils , M. Schlesner , Bioinforma. Oxf. Engl. 2016, 32, 2847. 10.1093/bioinformatics/btw313 27207943

[59] E. Picardi , G. Pesole , Bioinformatics 2013, 29, 1813. 23742983 10.1093/bioinformatics/btt287

