# Supplementary material for: Porous Silicon Nanoneedles Efficiently Deliver Adenine Base Editor to Correct a Recurrent Pathogenic COL7A1 Variant in Recessive Dystrophic Epidermolysis Bullosa
Source: Adv Mater. 2025 Mar 12;37(17):2414728. doi: 10.1002/adma.202414728 (PMC12038538; doi:10.1002/adma.202414728)
Supplement: Supplementary file 1 — Supporting Information [file ADMA-37-2414728-s001.pdf]

ADVANCED MATERIALS

Supporting Information

for *Adv. Mater.*, DOI 10.1002/adma.202414728

Porous Silicon Nanoneedles Efficiently Deliver Adenine Base Editor to Correct a Recurrent Pathogenic *COL7A1* Variant in Recessive Dystrophic Epidermolysis Bullosa

*Salman Ahmad Mustfa, Marija Dimitrievska, Cong Wang, Chenlei Gu, Ningjia Sun, Katarzyna Romańczuk, Pawel Karpinski, Łukasz Łaczmański, John A. McGrath, Joanna Jacków-Malinowska and Ciro Chiappini**

Supporting Information

Porous Silicon Nanoneedles Efficiently Deliver Adenine Base Editor to Correct a Recurrent Pathogenic *COL7A1* Variant in Recessive Dystrophic Epidermolysis Bullosa

Salman Ahmad Mustfa[1], Marija Dimitrievska[1,2]*, Cong Wang[1,3], Chenlei Gu[1,3], Ningjia Sun[1], Katarzyna Romańczuk[4], Pawel Karpinski[5], Łukasz Łaczmański[4], John A. McGrath[2], Joanna Jacków-Malinowska[2] and Ciro Chiappini[1,3]*

*Equal Contribution

Affiliations

1. Centre for Craniofacial and Regenerative Biology, King's College London, London, United Kingdom
2. St John's Institute of Dermatology, School of Basic & Medical Biosciences, King's College London, London, United Kingdom
3. London Centre for Nanotechnology, King's College London, London, United Kingdom
4. Hirszfeld Institute of Immunology and Experimental Therapy, Polish Academy of Sciences, Wroclaw, Poland
5. Department of Genetics, Wroclaw Medical University, Wroclaw, Poland

Figure S1. Bystander editing near target site following nanoneedle editing

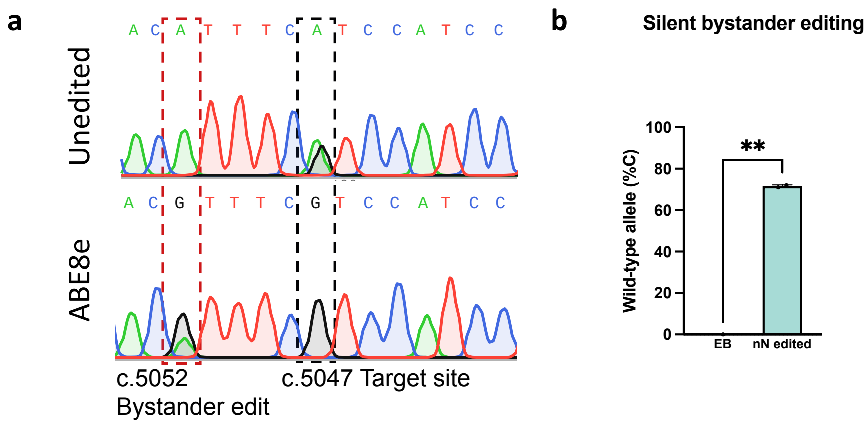

Figure S1. Bystander editing
a) Sanger sequencing chromatograms showing the A>G bystander variant introduced at c.5052 (red dotted box) in ABE8e successfully nanoneedle edited primary RDEB fibroblasts. **b)** Quantification of bystander editing using next generation sequencing shows 71.5% editing in ABE8e nanoinjected cells (n = 2) compared to untreated cells (n = 1). Data are presented as mean values ± S.D. Statistical significance tested using an unpaired students t-test. **: $p < 0.01$; RDEB – recessive dystrophic epidermolysis bullosa.

Figure S2. Differential gene expression profile of RDEB and nanoneedle edited fibroblasts

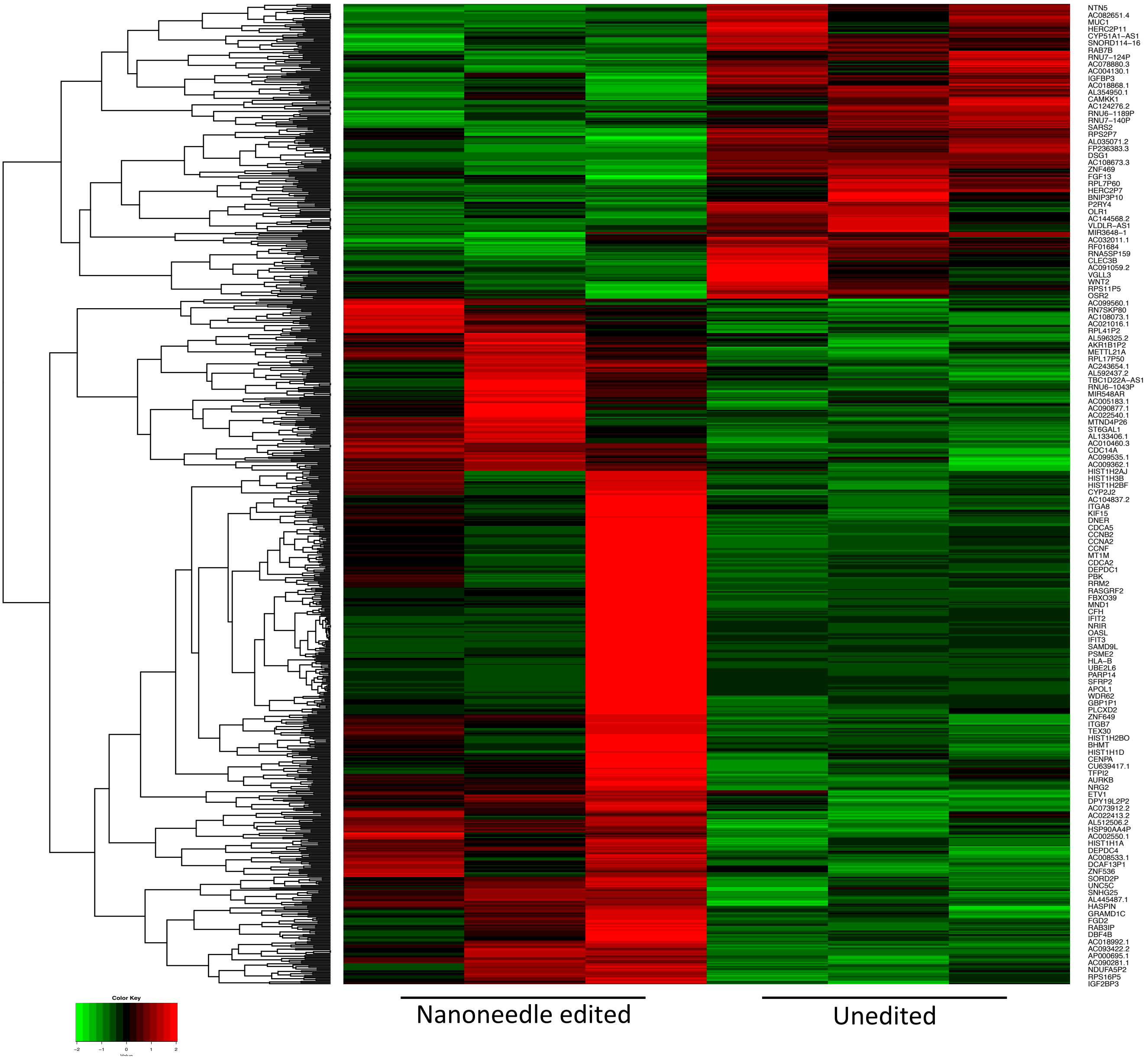

Figure S2. Differential gene expression profile of RDEB and nanoneedle edited fibroblasts

Heatmap of all differentially expressed genes in nanoneedle edited and unedited primary RDEB fibroblasts

Figure S3. Uncut and unadjusted cell lysate Western blots

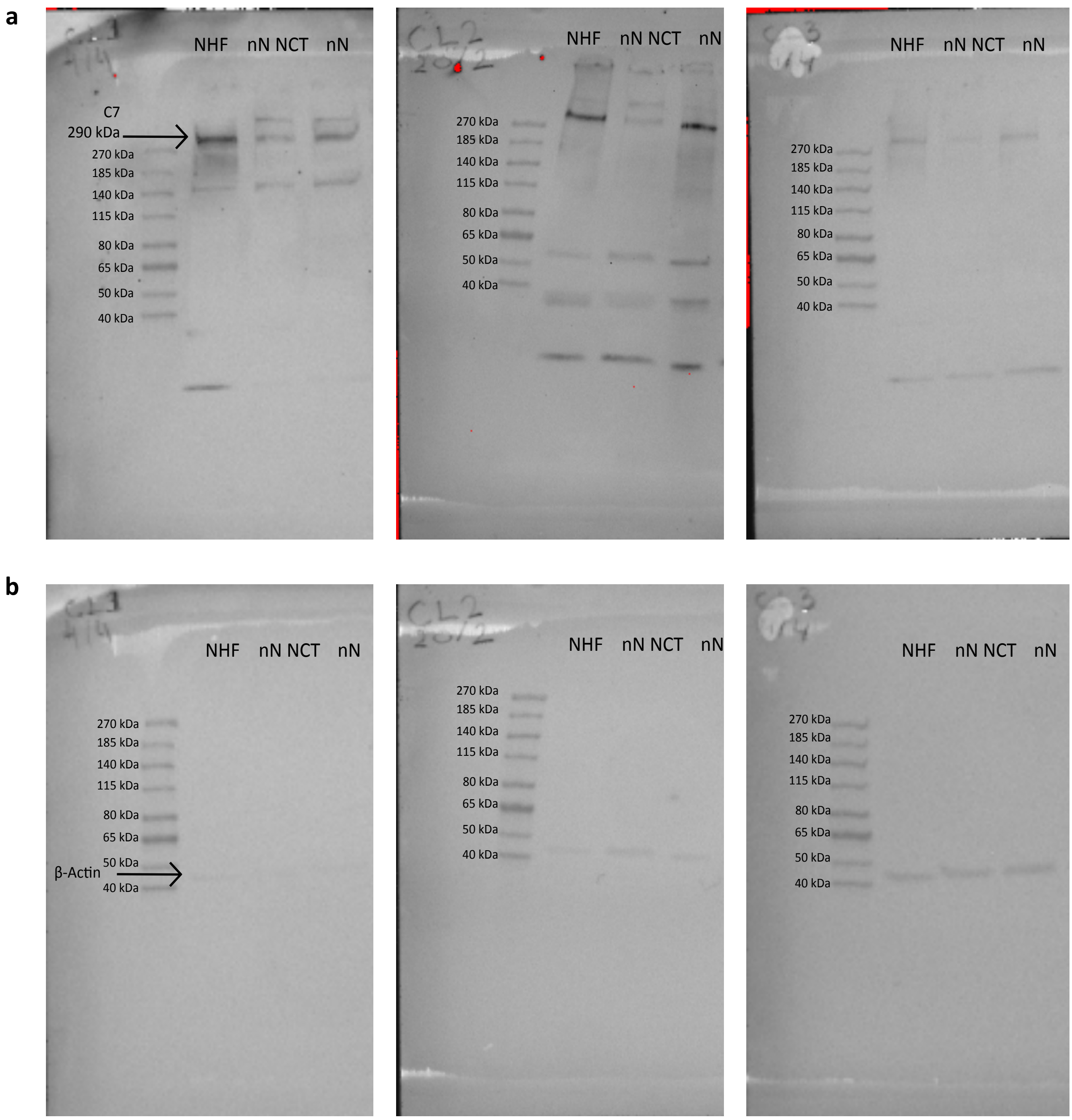

Figure S3. Uncut and unadjusted cell lysate Western blots
Total cell lysate blots from healthy primary fibroblasts (NHF), unedited (Nn NCT) and nanoneedle edited primary RDEB fibroblasts (nN) probed with a) anti-type-VII collagen polyclonal antibody and b) anti-β-actin. Three panels show three independent biological replicates.

Figure S4. Uncut contrast adjusted cell lysate Western blots

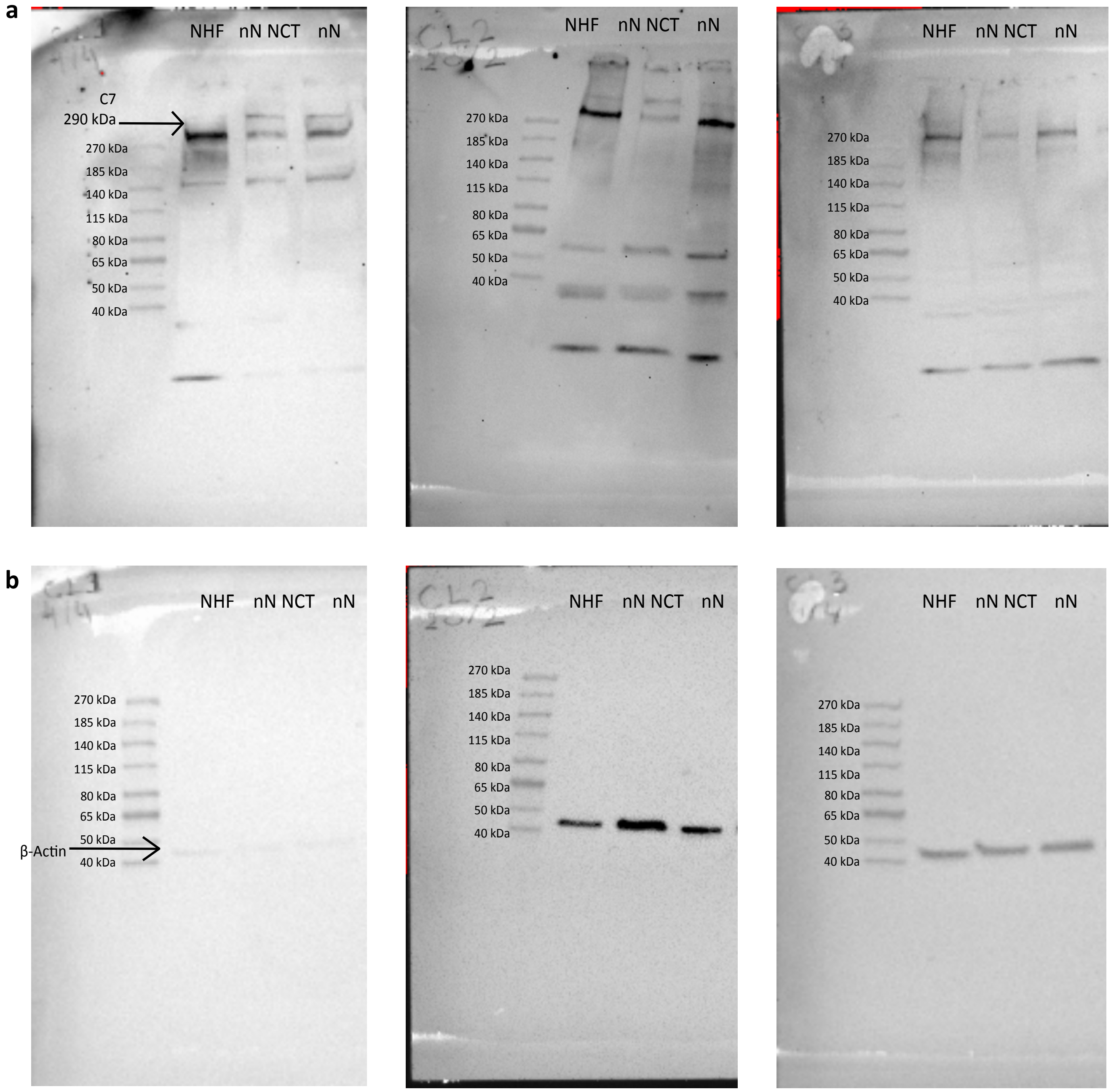

Figure S4. Uncut, contrast adjusted cell lysate Western blots
Contrast adjusted total cell lysate blots from healthy primary fibroblasts (NHF), unedited (Nn NCT) and nanoneedle edited primary RDEB fibroblasts (nN) probed with **a)** anti-type-VII collagen polyclonal antibody and **b)** anti-β-actin. Contrast adjustment with the same parameters was performed to panel a and b from each replicate. Three panels show three independent biological replicates.

Figure S5. Cell medium Western blots

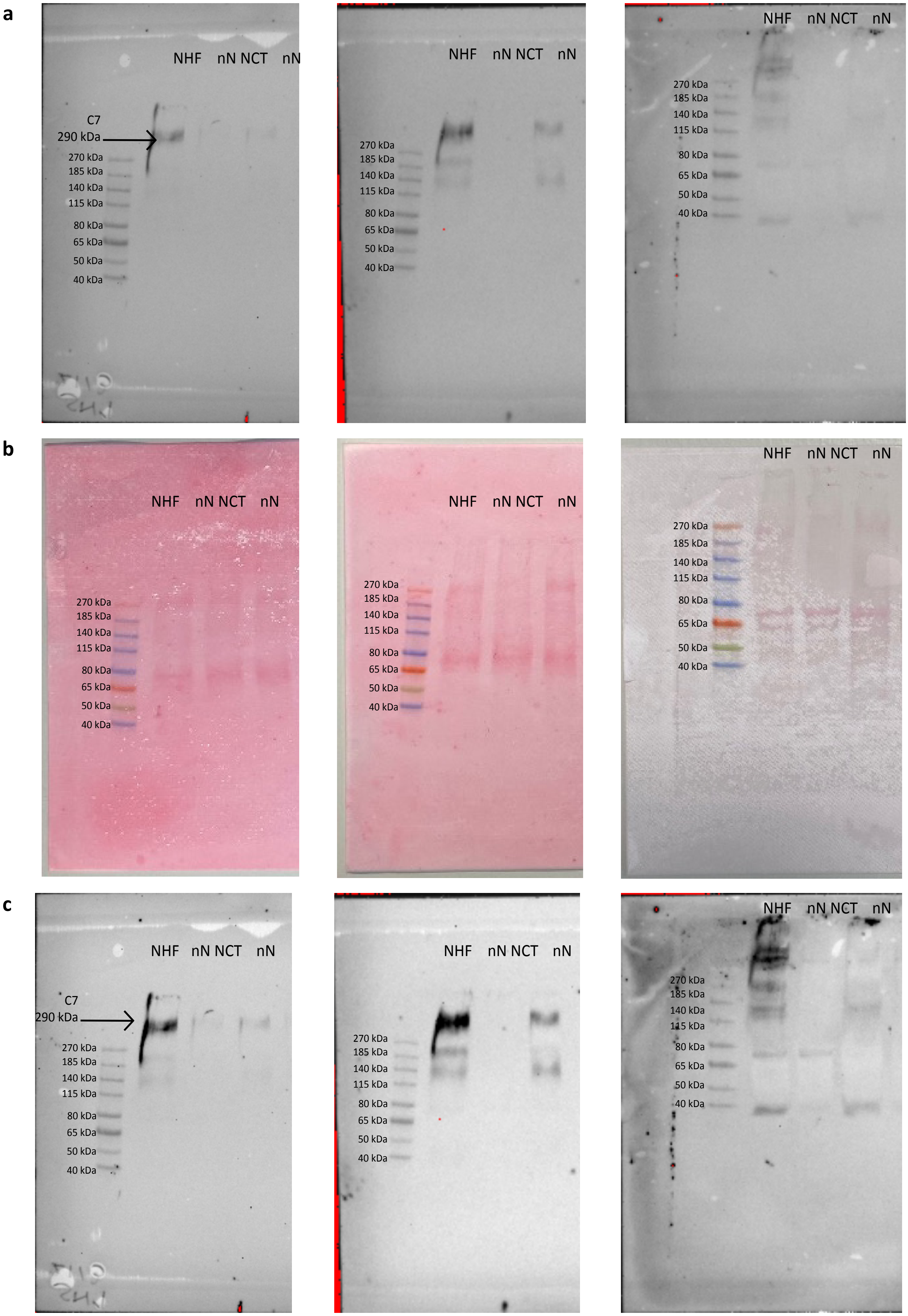

Figure S5. Cell medium Western blots
Uncut and unadjusted total cell medium secreted proteins blots from healthy primary fibroblasts (NHF), unedited (Nn NCT) and nanoneedle edited primary RDEB fibroblasts (nN) probed with **a)** anti-type-VII collagen polyclonal antibody. **b)** Ponceau stain for total protein. **c)** Contrast adjusted blot probed for type VII collagen. Three panels show three independent biological replicates.

Table S1. List of off-target sites

OT site	Fw primer 5'- 3'	Rv primer 5'- 3'	Target name	Target gene	Target amplicon 5'-3'	Target seq 5'-3'
OT-1	CGAGATGATAGGGAGGCAAAG	TGTTTGTGGTGGACTCGGC	NM_001317948.2 Homo sapiens ADP ribosylation factor like GTPase 10 (ARL10), transcript variant 1, mRNA	ARL10	GTGGATGTGCTGGTGTTTGTGGTGGACTCGGCTGACCGACTGCGGCT<br>GCCCTGGGCCCGACAGGAGCTGCACAAGCTGCTGGACAAGGACCCT<br>GACCTGCCTGTCGTCGTGGTGGCCAACAAGCAGGTGAGGGCTGTGA<br>GAGGGCAGCTCGGTCCAGGTGACATCCACTCAGAGATGCTTGGGCAG<br>GGGCAAGGAGCGCTGCCTGGGCCCATGGCCTGGAGAGGGTGGCTGA<br>GATGCTGCCCCCACCTCTTCTACCTATGCCCTGTCCTGATCCCTGCCTCT<br>GTCTTCCTTCCTCTTTGCCTCCCTATCATCTCGTACTCTGGAGAGA	GCATCTCAGCCACCCTCTCCAGG
OT-2	CAAACTCCCCTGCTGACCTC	CCCTCCCTGCAGATTCCAAG	NM_177403.6 Homo sapiens RAB7B, member RAS oncogene family (RAB7B), transcript variant 1, mRNA	RAB7B	AGCTCACTGCAAGGAGTCTCCCTCCCTGCAGATTCCAAGGCTGGAATC<br>TTTTTCTTCTGGCTCCAGGCAGCACAGACAGGGCCTAGCCTGGAGAG<br>GGTGGACAAGATGTCCTCTCAGGGTCTTCAATGGCCAAGTCCATGCCC<br>ACTGCAGAATCTTTCTGTCTACCCGTAAGTATCCTTTCCTGAGTTCCAG<br>GCAAAGCTGGGGATGTTAGCCTATGACTGTCATCTGACTTGGAAGGTA<br>CACCTAGGGGCCGGGGGGAGGTCAGCAGGGGAGTTTGGGAGCCAC<br>TTCTCCCCCCACGTGGCAC	ACATCTTGTCCACCCTCTCCAGG
OT-3	GCGTCTGTAGAGCCGATACC	CCCACTTTTCCCAGGCATT	NM_001370326.1 Homo sapiens ankyrin repeat and fibronectin type III domain containing 1 (ANKFN1), transcript variant 3, mRNA	ANKFN1	CTCAACACACCTAACAGCGAGGGCTTGACACCCCTGGATATTGCCATC<br>ATGACCAACAATGTGCCCATTGCAAGGATTCTTCTGAGGACAGGGGC<br>CCGAGAAAGTCCACACTTTGTCAGCCTGGAAAGCCGAGCAATGCACC<br>TCAACACACTGGTCCAGGAAGCCCAGGAGAGGGTGAGTGAACTGTC<br>TGCCCAGGTGGAGAATGAAGGATTCACTCTGGACAACACAGAGAAA<br>GAGAAGCAGCTGAAAGCTTGGGAGTGGAGGTATCGGCTCTACAGAC<br>GCATGAAAACAGGCTTTGA	ACAGTTCACTCACCCTCTCCTGG
OT-4	GGGGGATGAGGGCAGAATTT	TTTGGGGGTCCAGGAGGAAT	Homo sapiens chromosome 5 clone CTD-2207L2, complete sequence	ADAMTS12	AAGGTGACATCAGAAGTGTACATGTACCTGGGGGTGGGGGATGAGG<br>GCAGAATTTGAGGGCAGAGACCGTGAAATAACATTTGAATCATCCTCT<br>CCTGGGCCTAAAGCAACACAAGCTCTCTCTTAGACCCCTGAATTTTTCT<br>GAGTTTATATTTCAGATGAATGTGAAAAACATTTTTAAAAACATTCCTAT<br>ATTGCCACAAAACCTCCAGATAAAGCTGCAGGTGTATAGCTGATGAGA<br>GATGGACTTACTTAAAGCTCCTGCTCTGTCCTCGGAGGAGCTGAATCA<br>CGGGCAGTTACAGATTCCTCCTGGACCCCCAAACAGTGTGCAAGTTAC<br>AAATTAATAAATAGTGAGAAAAAATGGAGTTTTCCCCAATGTTTCCCAG<br>TAGATCCCAGAAAA	ACATTTGAATCATCCTCTCCTGG
OT-5	TGGTTCCCGGTTGTCTATGG	GAGTCTTGTGGAAGGTCTTTA	Homo sapiens chromosome 5 clone RP11-53K6, complete sequence	SPOCK1 intergenic	GATTACATTATACTGTGTTCACCCATTTGAGAAATCTTTGCTGGTTCCCG<br>GTTGTCTATGGGATAGAATCTAAAAGACTGTCATCCAGATCTCATCTTA<br>AATATGACCACATACTTCACCCAACCTCTCCTGGCCCATTTCCTTTCAAG<br>GTCTGTAACCCTCACTGCATCCCTAGGCCTTTCTACCACATGTCCCTGTT<br>TTATTCTCTTGATAGTATTACTGATTTTCTTATTTATTCACCTATCTTGCTT<br>ACTGCTGTATCCCAGTACTTAAACCATACCTGTTATGTAGTGTTGTCCTC<br>TGCAATTGTGGATGACTTGGCTTGAAATTAAAGACCTTCCACAAGACT<br>CTTTTGGGAACGAAATTATTCCCAAGAGACATT	ATACTTCACCCAACCTCTCCTGG

OT-6	CTACTCAGGAGGCTGAAGC	AGGGCAGGTGAAAGGAAGGC	Homo sapiens BAC clone RP11-275G11 from 7, complete sequence	GSAP intron	AAAAAGTTTTAATTAACCAGACATGATGACATGTACGTGTAGTCCTAGCTACTCAGGAGGCTGAAGCAAGAGGACTGCTTGAGCCCAGGAGTTCAAGGTTGCAGTGAGCTGTGACTATGTCATTGCACTCCAGCCTGGGCAACAGAGTGAGACCCTACCTCAAAAAAAGAAAAAAAAAAAAAGTATGACATACTTATTGTGGTGTTTTGTATGTATTAATTTTCATCCACCCACTCCTGGTTCACAATGCCCATAGCCTTTGTTACAGCCTGATGTTATAATATCGGGTATTTTAGGCTTCGGGACCAGGACTAGTCTCGGGAAACTCTCTCTCCTGCCTTCCTTTCACCTGCCCTAAGGCATGACTCTCCCCTCCACC	AATTTTCATCCACCCACTCCTGG
OT-7	CTCATAACTCCCACAACAGG	AGCCAGCAACATTGACCTCT	Homo sapiens BAC clone RP11-318K6 from 2, complete sequence	AC009410.1 - AC074019.1 intergenic	TCAGAAAACCTCTCCTGATACTGAACCAAAGTTTACTTCCTCATAACTCCCACAACAGGTATTAGTTCACCTGCCAGGACCACAGATGACTAATCTAGTCTTTCCACATGACAGTTTTTCAAATATTTGAAGGCAAAACTTCTATCTCCCATGATCCTTCTCTTAAGGGAAAATACCTCCACATCCCTTAAGAATTAAAAAAAGGTCTTCAGAGAACATGATTTTGAATTCCCTCACCATCCCATCCATCCTCTTCTGGATAATTTATTTATAAATTTCTTCAACTGTTTTGGTTTCAGAATAGAGGTCAATGTTGCTGGCTTAATCTTATTTCTCTACTGCAGATGTGTTCCTTTAAACAAGAGGTTTAGTTTACTTAAAACT	CCATCCCATCCATCCTCTTCTGG
OT-10	CCCAGAGGATCACCTTTCCC	CAACCCTGAGAGACAGGTGC	Homo sapiens BAC clone RP11-364J6 from 2, complete sequence	DNER intron	TTTGTCAATTATCCTTGTACATTCGTTATCTAATTTAAGCTGACAAACAACCCTGAGAGACAGGTGCTGCTAGGAGGATGCCTACCCCATCCTCCAGGTGAGGATGGTTGCAATATATAGTCAGACAGGAGAAGGAATTAACAGCAGGATGCAAGAAGTTGCCTGGGTGCATTGGACTGGGATGGGTAGATTCTAAACTTGAGCTCAGGCCTTTGAATTGAGCTGCAAGGTGGAACATTATGTCAGCACACGAAGGCACCAGAGTTCCAAGTTCCAACCCCAAAGGTACTATATGCTGGATGAGAAACAAAGACAACTGAAAGGACACAGAATGCTAAACTAGGGGAAAGGTGATCCTCTGGGAACTGTCCAAGTGTCCAAGCAAGAGGCAGTGGGTCGGG	ATATTGCAACCATCCTCACCTGG
OT-11	TCACACCCAGGAATGGAGCC	AGGCAAGGGAAACTTAGGCAA	Human DNA sequence from clone RP5-884C9 on chromosome 1p34.1-35.3, complete sequence	RP5-884C9.2-LINC01343 intergenic	AATAAAACCTATGTCTTTGGGTTATTGTAAAGATTAGGCAAGGGAAACTTAGGCAAAATCTCTTTGTCATCTGAGAGGGGATGTCCATTTAATTGGCATGTCAGTAGGGCAGGCATATTGTCCATTTCACAGGTGAGGAGCTTGAGGCCTGGAGAGGGTGGATGACCTGAAGAAGAAGCTGGCAGCATCACCAGGCAGGGCTGCATGGCAACTGGGGCTGCTTCTCTCTCTGCCACTACAGGATCCTGGCTCCCTCACACCACCCCCATGCTGATGCCCCACACGGGCTGCAACAAGCCCTGCTGGCCTGGCTCCATTTCTGGGTGTGAAATTTGGCTGAGTCGGCACCTGGTAGAATGGAGCAATTGCTATCCTGGAGCCTGGGGCCTGGGACAGGGCCAATGCGCAGACCAAGGGTTGATGGGCGGATCTGACCAAGGGTTGGTGGGTGGGCCTGGCGGGCTTGGCCAGGGAACTCGCCAAAGCT	TCAGGTCATCCACCCTCTCCAGG

Table S2. List of off-target single nucleotide variants detected outside the editing window

Sample	Off-target	Position	Gene	Change	Mutation type	Nr Alt reads	Nr Ref reads
N2	6	7:100010457	LOC105375423	CAAAAAAAAAAAAAAAAGGTGGC>CAAAAAAAAAAAAAAGGTGGC,CAAAAAAAAAAAAAAGGTGGC	Deletion	44	19
N2	6	7:36418868	ANLN	c>t	Substitution	287	0
N2	6	7:36418376	ANLN	t>a	Substitution	278	0
N2	6	7:36418360	ANLN	g>a	Substitution	269	0
N2	6	7:100010360	GSAP	GAAAAAAAAAAAAGTATG>GAAAAAAAAAAAGTATG	Deletion	258	0
N2	6	7:100010934	ZKSCAN1	c>t	Substitution	247	0
N2	6	7:100011033	ZKSCAN1	tca>tcct	Insertion	232	1
N2	6	7:100011044	ZKSCAN1	tccc>ccct	Insertion	229	0
N2	6	7:36418754	ANLN	CTTTTTTTTTTTTGTCTCA>CTTTTTTTTTTTGTCTCA	Deletion	57	69
N3	4	5:33770369	ADAMTS12	t>c,g	Substitution	81	86
N3	4	5:33770360	ADAMTS12	t>c,g	Substitution	76	62
N3	4	5:33770374	ADAMTS12	c>a,t	Substitution	61	62
N3	4	5:33770366	ADAMTS12	a>g	Substitution	139	36
N3	4	5:33770365	ADAMTS12	a>g	Substitution	121	12
N3	4	5:33770355	ADAMTS12	a>c	Substitution	103	11
N3	4	5:33770372	ADAMTS12	a>g	Substitution	98	55
N3	4	5:33770361	ADAMTS12	t>g	Substitution	94	87
N3	4	5:33770362	ADAMTS12	a>c	Substitution	87	12
N3	4	5:33770367	ADAMTS12	t>c	Substitution	78	77
N3	4	5:33770373	ADAMTS12	a>g	Substitution	74	85
N3	4	5:33770375	ADAMTS12	TTGCACACT>TAAGAGACA	Substitution	21	0

Sample	Off-target	Number of out of frame mutations in each sample
N3	4	12
N2	6	9

Table S3. List of the top 55 differentially expressed genes in nanoneedle edited and unedited primary RDEB fibroblasts

Gene name
VAMP7
IL17RB
LSM14A
THBS1
NPIPP1
RAB13
RSPO3
BRD2
ZFP36
SCRIB
KRT1
SFRP4
MTATP6P1
SYNPO2
PODN
FTH1P11
FTH1
CHRM2
SCARNA7
ADM
GABARAP
FTH1P7
IGFBP3
AC064799.1
RPL7AP6
RPL41P1
AC090498.1
AC138969.1
FAM120B
SNORD3C
INTS3
SYNGAP1
FP671120.1
ABCC1
BAG6
CLIC4P1
RNA5−8SN3
HIST1H2AB

CUZD1
ST8SIA5
HIST1H2BB
HIST1H1B
HIST1H2AL
HIST1H2BL
RTP4
GTSE1
NDC80
RANBP17
KIF14
KNL1
CKAP2L
TTK
AC003092.1
RTKN2
HMMR
